# Transcriptomics reveals amygdala neuron regulation by fasting and ghrelin thereby promoting feeding

**DOI:** 10.1126/sciadv.adf6521

**Published:** 2023-05-24

**Authors:** Christian Peters, Songwei He, Federica Fermani, Hansol Lim, Wenyu Ding, Christian Mayer, Rüdiger Klein

**Affiliations:** ^1^ Department of Molecules–Signaling–Development, Max-Planck Institute for Biological Intelligence, 82152 Martinsried, Germany.; ^2^ Laboratory of Neurogenomics, Max-Planck Institute for Biological Intelligence, 82152 Martinsried, Germany.

## Abstract

The central amygdala (CeA) consists of numerous genetically defined inhibitory neurons that control defensive and appetitive behaviors including feeding. Transcriptomic signatures of cell types and their links to function remain poorly understood. Using single-nucleus RNA sequencing, we describe nine CeA cell clusters, of which four are mostly associated with appetitive and two with aversive behaviors. To analyze the activation mechanism of appetitive CeA neurons, we characterized serotonin receptor 2a (Htr2a)–expressing neurons (CeA^Htr2a^) that comprise three appetitive clusters and were previously shown to promote feeding. In vivo calcium imaging revealed that CeA^Htr2a^ neurons are activated by fasting, the hormone ghrelin, and the presence of food. Moreover, these neurons are required for the orexigenic effects of ghrelin. Appetitive CeA neurons responsive to fasting and ghrelin project to the parabrachial nucleus (PBN) causing inhibition of target PBN neurons. These results illustrate how the transcriptomic diversification of CeA neurons relates to fasting and hormone-regulated feeding behavior.

## Teaser

Central amygdala neuron diversity and the roles of appetitive amygdala neurons in hormone-regulated feeding behavior are unveiled.

## INTRODUCTION

Central nervous system regulation of feeding behavior controls basic energy needs and satisfies the pleasure associated with eating in processes called homeostatic and hedonic feeding, respectively. The classical view is that brain circuits controlling homeostatic feeding are mainly located in the hypothalamus, whereas hedonic feeding is controlled by the limbic and reward systems, including hippocampus, amygdala, prefrontal cortex (PFC), nucleus accumbens (NAc), and ventral tegmental area (VTA) (*1*, *2*). A more recent view is that the brain circuits controlling homeostatic and hedonic feeding are both active whenever food is consumed. The relative involvement of each circuit may depend on the palatability of food and the physiological state of the animal (*1*, *3*).

Here, we focus on the central amygdala (CeA), an evolutionarily ancient subcortical structure that controls emotional processing and promotes defensive and appetitive behaviors (*4*), including food intake and reward processing (*5*–*8*). The CeA consists exclusively of γ-aminobutyric acid–releasing (GABAergic) neurons that can be divided into subpopulations based on their molecular, electrophysiological, and functional properties. They are organized in reciprocally inhibitory microcircuits in three subregions of the CeA: the central capsular (CeC), the central lateral (CeL), and central medial subregions (CeM). Our current understanding of CeA cell diversity, anatomy, and function is incomplete, because molecularly defined CeA populations can show substantial overlap and may have different functions in the different CeA subregions (which are difficult to target by stereotactic injections), and their roles can differ from CeA neurons that were characterized on the basis of electrophysiological or anatomical properties (*9*, *10*). A deep analysis of the cell types, their distribution, and roles in CeA-controlled behavior is therefore needed.

Several molecularly defined CeA neuron types have previously been linked to food intake and reward behavior (*11*). Neurons expressing protein kinase Cδ (CeA^PKCδ^, also known as Prkcd) suppress feeding, in response to anorexigenic cues (*7*). In contrast, serotonin receptor 2a (Htr2a)–expressing neurons (CeA^Htr2a^) induce feeding by increasing the palatability of food (*6*). These neurons were shown to be required for consumption of palatable food by ad libitum fed animals, or after an overnight fast and a short post-fast feeding period, when the homeostatic needs were partially satisfied. CeA^Htr2a^ neurons partially overlap with somatostatin-positive neurons (CeA^Sst^) and other CeA populations marked by expression of *corticotropin releasing factor* (*Crh*) and *tachykinin 2* (*Tac2*) (*6*, *12*, *13*). CeA^Sst^ neurons promote not only reward behavior but also fear learning (*8*, *10*, *14*–*16*). Neurons expressing prepronociceptin (CeA^Pnoc^) that also partially overlap with CeA^Sst^ neurons mediate palatable food consumption and reward, similarly to CeA^Htr2a^ (*5*, *6*). Neurons expressing neurotensin (CeA^NTS^) promote reward behavior and the consumption of palatable fluids (*17*).

What has been an open question in the field is how appetitive CeA neurons become activated. They could be activated via their afferent inputs, some of which have been characterized anatomically, but not functionally, including the basolateral amygdala (BLA), hypothalamus, substantia nigra, parasubthalamic nucleus, and VTA (*6*, *8*, *18*). In addition, appetitive CeA neurons may be under hormonal control. One key hormone controlling feeding is ghrelin, a 28–amino acid peptide synthesized and secreted by gastric oxyntic cells (*19*). Ghrelin release increases while fasting and mediates its orexigenic effects through hypothalamic and extrahypothalamic regions, the latter including the hippocampus, amygdala, and VTA (*20*–*22*). This peptidic hormone increases appetite and food intake through activation of the growth hormone secretagogue receptor (GHSR) (*23*), and through opioid and dopamine receptors, favoring food consumption by enhancing the rewarding and incentive responses to food-related cues (*24*–*29*).

Ghrelin receptor expression is widespread including in the CeA (*30*, *31*). Functional effects of ghrelin on CeA neurons remain largely unexplored, except for evidence that ghrelin increases the amplitude of evoked inhibitory postsynaptic potentials (IPSPs) and the frequency of miniature inhibitory postsynaptic currents (mIPSCs) in rat CeA (*30*). Considering that CeA only contains GABAergic neurons (*32*), ghrelin could be increasing the inhibitory neurotransmission activating one or more subpopulations of neurons in the CeA.

Here, we present a transcriptomic taxonomy of adult mouse CeA cell clusters. We find four cell clusters associated with presumed orexigenic and two with anorexigenic activities. CeA^Htr2a^ neurons previously shown to promote feeding comprise three presumed appetitive clusters. Using electrophysiology and in vivo calcium imaging, we show that CeA^Htr2a^ neurons are activated by the presence of food, by fasting and ghrelin, and that the activity of these neurons is required for ghrelin’s orexigenic effects. Moreover, fasting and ghrelin activate CeA neurons projecting to the parabrachial nucleus (PBN), a region of the brain that has been described previously as important for feeding regulation (*6*, *33*, *34*). CeA→PBN projecting neurons inhibit their target PBN neurons and thereby increase feeding. Last, fasting of the animals results in dynamic gene expression changes in specific CeA clusters associated with synaptic transmission and spine growth. These findings enhance our understanding of cell type diversity in CeA and the role of CeA cell clusters in the hormonal regulation of food consumption.

## RESULTS

### Single-nuclei transcriptomic characterization of CeA neuron types

**Fig. 1.**
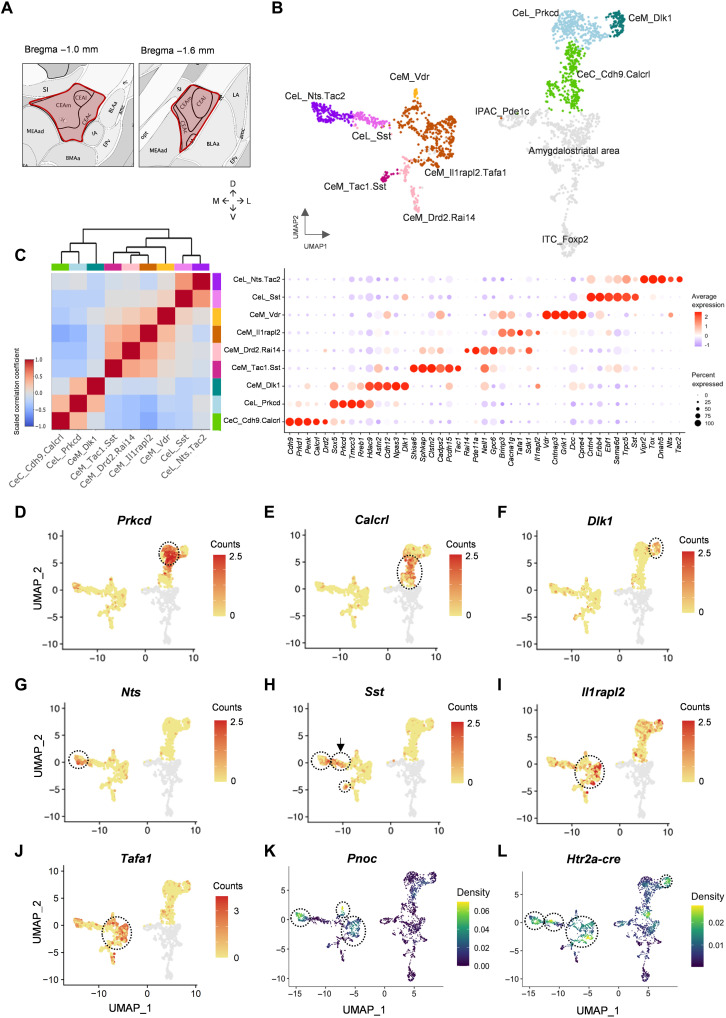
Transcriptomic cell type taxonomy of the mouse central amygdala. (**A**) Scheme showing the sampled central amygdala (CeA) regions highlighted in red. (**B**) Uniform Manifold Approximation and Projection (UMAP) representation of all inhibitory neurons of the CeA colored by cluster. Cells colored in gray are inhibitory neurons from regions outside the CeA, including the posterior limb of the anterior commissure (IPAC), the amygdalostriatal area, and intercalated cells (ITC). (**C**) Transcriptomic taxonomy tree (left) and molecular signatures of nine CeA cell clusters (right). Hierarchical clustering on Pearson’s pairwise correlation coefficients based on the mean expression of 2000 highly variable genes (HVGs) in each cell cluster. Molecular signatures of clusters by percentage of cells expressing the gene (circle size) and average gene expression (color scale). (**D** to **J**) UMAP plots showing PKCδ (Prkcd), Calcrl, Dlk1, Nts, Sst, IlRapl2, and Tafa1 clusters. Clusters are indicated by stippled circles. Arrow in (H) indicates the CeL_Sst cluster. (**K** and **L**) UMAP density plots of *Pnoc* (K) and *mCherry* (L) transcripts. The viral expression of *mCherry* is driven by Htr2a-Cre. Circles in (K) and (L) indicate cell clusters containing Pnoc and Htr2a-Cre–positive cells.

We used single-nucleus RNA sequencing (snRNA-seq) to identify the diversity of subtypes present in subregions of the CeA (Fig. 1A). Two datasets were obtained from 8-week-old naïve mice. A third dataset came from Htr2a-Cre transgenic mice expressing AAV-DIO-mCherry to determine which subtypes were included in the CeA^Htr2a^ population. We obtained 3325 single-nuclei transcriptomes (with a median of 3833 genes per nucleus) and performed principal components analysis (PCA) on the scaled gene expression data, followed by Uniform Manifold Approximation and Projection (UMAP) visualization and unsupervised clustering. We obtained 16 clusters and assigned them manually into cell classes based on the coexpression of multiple marker genes (fig. S1, A and C). As expected, GABAergic neurons were the predominant cell type (fig. S1B) forming 11 clusters (including two BLA interneuron clusters marked by *Nkx2.1*). A minor fraction (0.9%) of neurons expressed the excitatory marker *Scl17a7*. To gain a higher level of detail, we reclustered 2488 GABAergic neurons (Fig. 1B). We identified clusters that did not belong to the CeA, including the interstitial nucleus of the posterior limb of the anterior commissure (IPAC; based on *Pde1c* expression), the amygdalostriatal area (based on *Rarb* expression), and intercalated cells (based on *Foxp2* expression) (fig. S1, D to F) (*35*, *36*). For the remaining nine clusters belonging to the CeA, we constructed a taxonomy tree (Fig. 1C) based on the correlation of the expression of highly variable genes (HVGs) across cell types.

The first division separated three clusters from the others, namely, PKCδ (Prkcd), cadherin-9/calcitonin receptor-like (Cdh9/Calcrl), and delta-like 1 (Dlk1) (Fig. 1, D to F). PKCδ neurons were previously shown to reside in the CeL and were well characterized for their anorexigenic and other aversive functions (*7*, *37*) but are also involved in appetitive/affective behavior (*38*, *39*). This richness of functions may be because PKCδ neurons can be found in other clusters, most prominently in the Cdh9/Calcrl cluster (from now on termed Calcrl) (Fig. 1, D and E), which has been shown to be enriched in the adjacent CeC region (*8*). Calcrl neurons (also termed CGRPR) and the CeC subpopulation of PKCδ neurons were previously shown to drive aversive learning (*8*, *40*). The Dlk1 cluster that we identified has not been characterized and appears to be enriched in the CeM region (14% of CeM neurons) (fig. S2, A and B).

The second division separated NTS/Tac2 and Sst-expressing cells from the remaining four clusters (Fig. 1, B, C, G, and H). NTS and Tac2-positive cells were previously shown to be enriched in CeL and to mediate appetitive behaviors (fig. S2, C and D) (*8*, *12*). Many NTS/Tac2 cells expressed *Sst* at low levels, but the highest Sst-expressing cells formed a separate cluster (Fig. 1, G and H). Other Sst-positive cells were found in the CeM (fig. S2, A and E). This may explain the range of functions attributed to Sst cells including appetitive/reward (*41*) and aversive behaviors (*14*, *42*, *43*). NTS neurons were previously found to promote consumption of ethanol and palatable fluids (*17*).

The remaining four clusters were all enriched in the CeM subregion as shown by the marker *calcium voltage-gated channel subunit alpha1 G* (*Cacna1g*) (fig. S2F). The largest cluster (59% of all CeM neurons) was marked by *interleukin 1 receptor accessory protein like 2* (*Il1Rapl2*) and *TAFA chemokine like family member 1* (*Tafa1*) (subsequently named Il1Rapl2; Fig. 1, I and J). Other markers for this cluster included *Htr1b*, *Nefm*, and *Ebf1* but were also expressed elsewhere (fig. S2, G to I). Two smaller CeM clusters were identified (together, they accounted for 23% of the CeM population). One was marked by *tachykinin 1* (*Tac1*) and *Sst*, and the other was marked by *Drd2/Rai14* (fig. S2, J and K). The Tac1/Sst cluster may promote appetitive behavior, since optogenetic activation of Sst cells of the CeM was previously shown to promote rewarding behavior (*8*). The Drd2/Rai14 cluster contained dopamine receptor 2–positive cells of the CeM (fig. S2K). This cluster is distinct from a larger cluster of Drd2 cells residing in the CeC overlapping with the Calcrl cluster (Fig. 1E and fig. S2K) (*8*), and the function of CeM^Drd2/Rai14^ cells remains to be investigated. The fourth CeM cluster (4% of CeM neurons) was marked by *vitamin D receptor* (*Vdr*) (fig. S2L) and remains to be functionally characterized. In summary, snRNA-seq identified nine CeA cell clusters across the three subregions: one cluster in CeC (Calcrl), three clusters in CeL (PKCδ, NTS/Tac2, and CeL-Sst), and five clusters enriched in CeM (IL1Rapl2, Tac1/Sst, Drd2/Rai14, Vdr, and Dlk1). Most clusters were found in the CeM, a subregion of the CeA that has received less attention than the CeL.

Since previous work had shown that CeA^Pnoc^ and CeA^Htr2a^ neurons promoted food intake and reward behavior by inhibiting neurons in the PBN (*5*, *6*), we hypothesized that these populations overlapped. We plotted Pnoc expression density on a UMAP and found that Pnoc neurons were mainly expressed at similar levels (10% of cells) in the presumed appetitive CeM^IL1Rapl2^ and CeL^NTS/Tac2^ clusters (and the small CeM^Vdr^ cluster; Fig. 1K). CeA^Htr2a^ neurons were mainly expressed (40 to 50% of cells) in the presumed appetitive CeM^IL1Rapl2^ and CeL^Sst^ clusters, somewhat less in the CeL^NTS/Tac2^ cluster (30 to 40% of cells), and the CeM^Dlk1^ cluster (Fig. 1L). Immunostaining against nociceptin, the protein encoded by the *Pnoc* gene, using Htr2a-Cre;tdTomato animals, confirmed that CeA^Pnoc^ and CeA^Htr2a^ neurons partially overlapped (54% of CeA^Htr2a^ neurons were nociceptin-positive, 34% of nociceptin-positive neurons belonged to the CeA^Htr2a^ population; see fig. S2M).

### Ghrelin activates CeA neurons

**Fig. 2.**
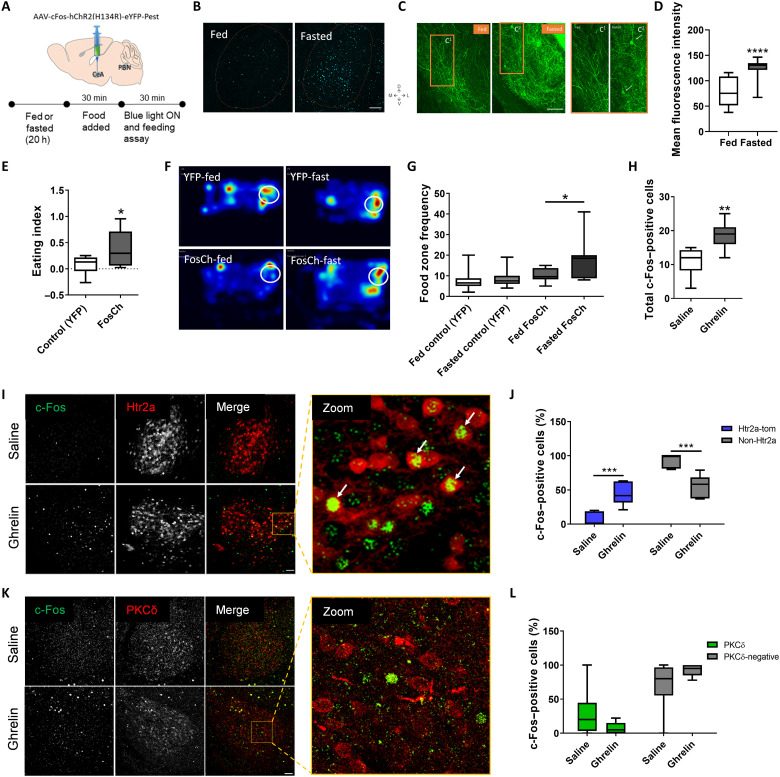
Fasting and ghrelin activate central amygdala (CeA) neurons. (**A**) Delivery of AAV-cFos-hChR2(H134R)-EYFP-Pest (FosCh) virus into CeA in wild-type mice and protocol of feeding assay. (**B**) c-Fos staining in CeA from fed and fasted animals. Scale bar, 250 μm. (**C**) Enhanced yellow fluorescent protein (EYFP) immunostainings in CeA from fed and fasted animals injected with FosCh virus. C^1^ and C^2^ are zoom insets from fed and fasted animals, respectively. Arrows in C^2^ indicate EYFP-positive CeA neurons. Scale bar, 250 μm. (**D**) EYFP mean fluorescence intensities in CeA from (C). *t* test; ****P < 0.0001; *n* = 17 sections from *n* = 3 animals per group. (**E**) Eating index [(fast − fed)/total consumption] after 30 min with blue light stimulation (20 Hz). *t* test; *P < 0.05; *n* = 6 animals per group. (**F**) Representative heatmaps of mouse dwell time after light activation in CeA (20 Hz, blue light stimulation for 30 min). White circles represent the food zones. (**G**) Frequency of the mice visiting the food zones [white circle in (D)]. One-way analysis of variance (ANOVA), *P < 0.05; *n* = 6 animals per group. (**H**) CeA c-Fos–positive cells per CeA section after 90-min intraperitoneal injection of saline or ghrelin (10 μg). *t* test, **P < 0.01; *n* = 7 sections from *n* = 3 animals per group. (**I**) Representative images of c-Fos staining (green) in the CeA of Htr2a-tomato (red) mice after 90-min intraperitoneal injections of saline or ghrelin (10 μg). Arrows indicate double-positive cells. Scale bar, 50 μm. (**J**) Percentage of c-Fos–positive cells in Htr2a and non-Htr2a neurons from (H). Two-way ANOVA, ***P < 0.001; *n* = 3 animals per group. (**K**) Representative images of c-Fos (green) and PKCδ (red) immunostaining in the CeA of wild-type mice after saline or ghrelin intraperitoneal injections. Scale bar, 50 μm. (**L**) Percentage of c-Fos–positive cells in PKCδ and non-PKCδ neurons. Two-way ANOVA, *n* = 3 animals per group.

To begin addressing the activation mechanisms of appetitive CeA neurons, we asked whether an overnight fast would result in activation of CeA neurons. Using the *immediate-early gene c-Fos* as a surrogate marker for neuronal activity, we found that the abundance of c-Fos–positive cells in the CeA increased in fasted animals (fig. S3, A and B). To evaluate the functional relevance of these behavioral activity-defined (c-Fos–positive) neurons, we tested whether photostimulation of these cells would lead to an increase in food uptake in the animals that had experienced the stimulus (fasting). We performed stereotactic injections of AAV-cFos-hChR2(H134R)-EYFP-Pest (FosCh) (*44*) in CeA (Fig. 2A). The FosCh virus expresses channelrhodopsin (hChR2) and enhanced yellow fluorescent protein (EYFP) under the c-Fos promoter and allows photoactivation of c-Fos–positive cells. We kept the animals at ad libitum feeding or subjected them to a 20-hour fasting period to induce expression of c-Fos and FosCh (Fig. 2, B and C). In the CeA of fasted animals, EYFP immunoreactivity indicating increased expression of ChR2-EYFP protein was significantly higher than in fed animals (Fig. 2D), and the majority of EYFP-positive cell bodies were immunoreactive for c-Fos (fig. S3, E and F). After fasting, all mice were given food for 30 min to reduce the contribution of fasting itself to food uptake. Thereafter, the CeA was photostimulated in the presence of food, and feeding-related behavior was evaluated. The results showed that FosCh-expressing animals subjected to fasting consumed more food than FosCh-expressing animals kept at ad libitum feeding, while there was no difference between fasted and fed control animals (fig. S3G). The eating index [(fast − fed)/total consumption] was higher in fasted animals expressing FosCh than EYFP control virus (Fig. 2E). Moreover, the frequency that mice went into the food zone was higher in fasted than fed FosCh animals (Fig. 2, F and G).

The levels of the hunger hormone ghrelin are known to increase during fasting (*45*), and we confirmed that ghrelin signaling is required for efficient feeding behavior after fasting (fig. S3C) (*46*). We therefore asked if ghrelin mediates the effects that fasting exerts on CeA neurons. Intraperitoneal injection of ghrelin at concentrations that led to a dose-dependent increase in food uptake (fig. S3D) also led to an increase of c-Fos–positive cells in the CeA in satiated animals (Fig. 2H). To study the effects of ghrelin on appetitive CeA neurons, we compared the effects of ghrelin in the CeA^Htr2a^ population that comprises three presumed appetitive cell clusters, versus CeA^PKCδ^ cells, the major aversive cluster. Quantification of c-Fos expression levels in the CeA showed that animals injected with ghrelin had a higher number of c-Fos–positive cells with a markedly higher increase among CeA^Htr2a^ neurons compared to Htr2a-Cre–negative neurons (Fig. 2, I and J, and fig. S3H). In contrast, CeA^PKCδ^ neurons did not show an increase in the percentage of c-Fos–positive cells after ghrelin application (Fig. 2, K and L, and fig. S3I), suggesting that the ghrelin effect in the CeA was cell type–specific.

### Activities of ghrelin-responsive CeA^Htr2a^ neurons increase during feeding

To better understand how appetitive CeA neurons modulate food intake, we performed in vivo Ca^2+^ imaging in freely moving mice. We confronted fed or fasted (20 hours) mice with food and/or injected systemically ghrelin and monitored the activities of CeA^Htr2a^ neurons expressing the calcium indicator GCaMP6m across different behavioral sessions (fig. S4, A and B). As observed previously (*6*), the majority of CeA^Htr2a^ neurons increased their activity during food consumption (fig. S5, A to D). We now found that, when mice were ad libitum fed, the presence of food increased the activities of the majority of CeA^Htr2a^ neurons, although food consumption was minor (fig. S4, C and E). In the fed state in the absence of food, exogenous ghrelin increased CeA^Htr2a^ neuronal activities (fig. S4, D and F). Whenever food was presented, neuronal activities did not increase further, because ghrelin administration alone already activated the neurons to a similar extent as food (fig. S4G). After ghrelin injection, in the presence of food, CeA^Htr2a^ neuronal activities strongly correlated with feeding. This was observed for the first feeding bout for individual neurons and for the population as a whole (fig. S4, H to J). It was also revealed by comparing the neuronal activities while the animals were feeding or not feeding during the entire session (figs. S4K and S5E). Together, these data revealed that ghrelin administration activated CeA^Htr2a^ neurons, even in the absence of food. In the presence of food, the activities of ghrelin-responsive CeA^Htr2a^ neurons increased with food consumption.

### Ghrelin directly increases the excitability of CeA^Htr2a^ neurons

**Fig. 3.**
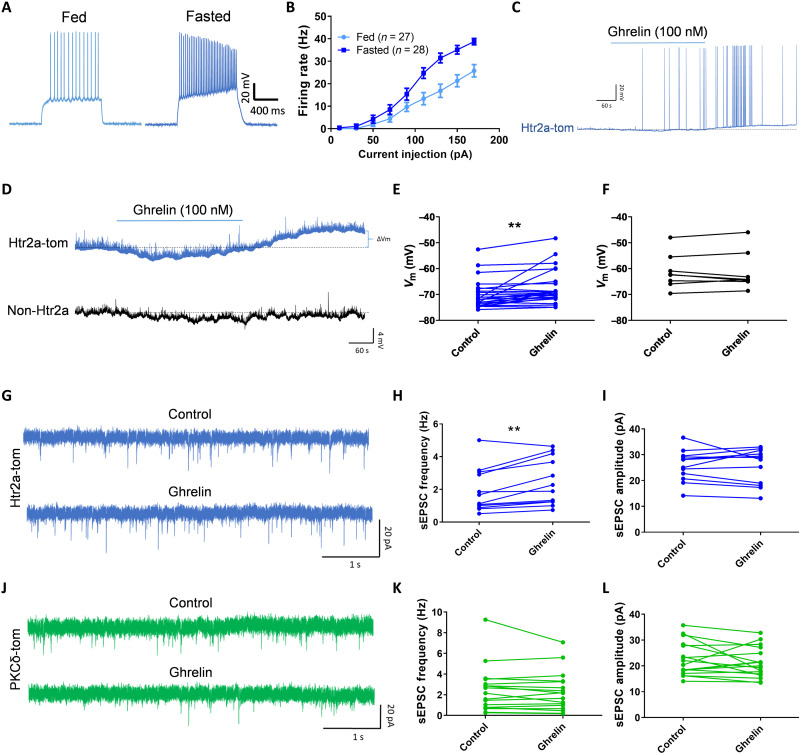
Ghrelin excites serotonin receptor 2a (Htr2a)–expressing neurons (CeA^Htr2a^). (**A**) Whole-cell current-clamp recordings of CeA^Htr2a-tom^ neurons from fed and fasted animals. (**B**) Firing rates (in hertz) after injecting different current steps in CeA^Htr2a-tom^ neurons of fed and fasted animals. *n* = 27 neurons for fed, *n* = 28 neurons for fasted. (**C**) Example of an Htr2a neuron that increased the firing after ghrelin application (100 nM, 3 min). (**D**) Whole-cell current-clamp recordings of CeA^Htr2a-tom^ and non–Htr2a-tom neurons, showing that ghrelin depolarized Htr2a-tom neurons after 3 min of ghrelin perfusion (100 nM). (**E** and **F**) Quantification of the membrane potentials before and after ghrelin perfusions from (D) of CeA^Htr2a-tom^ (E) and non–Htr2a-tom neurons (F). Paired *t* test, **P < 0.01; *n* = 26 neurons for CeA^Htr2a^, *n* = 8 neurons for non–Htr2a-tom. (**G**) Representative spontaneous excitatory postsynaptic current (sEPSC) recordings before and after ghrelin perfusion in CeA^Htr2a-tom^ neurons. (**H** and **I**) Quantification of sEPSC frequency and amplitude from (G). Paired *t* test, **P < 0.01; *n* = 12. (**J**) Representative sEPSC recordings before and after ghrelin perfusion in CeA^PKCδ-tom^ neurons. (**K** and **L**) Quantification of sEPSC frequency and amplitude from (J). Paired *t* test, *n* = 17 neurons.

We next investigated the mechanism of activation of appetitive CeA neurons by using electrophysiological recordings in brain slices. The results revealed that, in fasted animals, the excitability of CeA^Htr2a^ neurons increased significantly compared to fed animals (Fig. 3A). Current injections showed a higher firing rate in fasted than fed animals (Fig. 3B). To begin addressing whether the differences in action potential frequencies may be caused by increased levels of endogenous ghrelin, we performed current-clamp recordings in brain slices perfusing ghrelin for 3 min. The results showed that ghrelin (100 nM) induced the firing of CeA^Htr2a^ neurons (Fig. 3C), depolarizing most of them after a few minutes of ghrelin application (100 nM and 1 μM) (Fig. 3D and fig. S6, A and B). Significant differences in membrane potentials (*V*_m_) before and after ghrelin perfusion were observed in Htr2a-Cre;tdTomato, but not Htr2a-Cre–negative neurons (Fig. 3, E and F).

Voltage clamp recordings of spontaneous excitatory postsynaptic currents (sEPSCs) in Htr2a-Cre;tdTomato neurons (Fig. 3G) showed that ghrelin increased the frequency of excitatory neurotransmission (Fig. 3H), but not the amplitude (Fig. 3I). Conversely, we observed no ghrelin-induced changes in excitatory neurotransmission in CeA^PKCδ^ neurons (Fig. 3, J to L). These results suggest that ghrelin induces the release of glutamate specifically from presynaptic inputs to CeA^Htr2a^ neurons, thereby increasing the excitability of these presumed appetitive neurons.

### CeA^Htr2a^ neurons are necessary for ghrelin to increase feeding

**Fig. 4.**
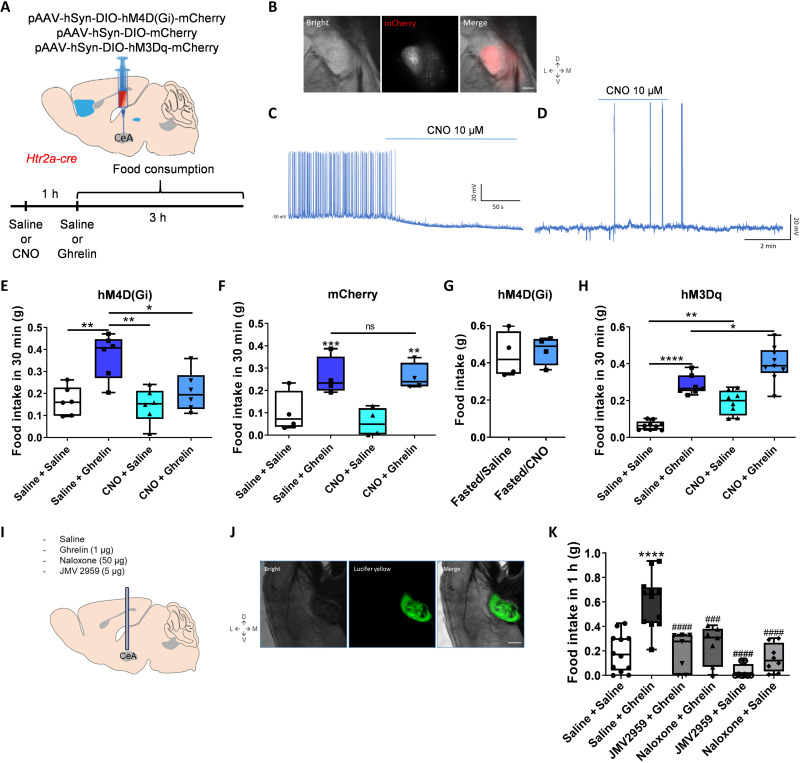
Ghrelin requires serotonin receptor 2a (Htr2a)–expressing neurons (CeA^Htr2a^) to increase food intake. (**A**) Delivery of pAAV-hSyn-DIO-hM4D(Gi)-mCherry, pAAV-hSyn-DIO-mCherry, or pAAV-hSyn-DIO-hM3Dq-mCherry viruses bilaterally into the CeA of Htr2a-Cre animals. After 4 weeks, saline or CNO (0.4 mg/kg) was injected intraperitoneally and after 1 hour followed by saline or ghrelin (10 μg). In the hM3Dq experiment, CNO (1 mg/kg) was used at a 30-min interval before saline/ghrelin injection. Food consumption was registered for 3 hours. (**B**) Representative CeA images showing the expression of mCherry virus in CeA^Htr2a^ neurons. Scale bar, 250 μm. (**C** and **D**) Whole-cell current-clamp recordings of CeA^Htr2a^ neurons. CNO (10 μM) inhibits neurons when hM4D(Gi) is expressed (C). CNO (10 μM) excites neurons when hM3Dq is expressed (D). (**E**) Food intake (30 min) in satiated Htr2a-Cre mice expressing hM4D(Gi) virus. One-way ANOVA, *P < 0.05 and **P < 0.01; *n* = 7 animals per group. (**F**) Food intake (30 min) in satiated Htr2a-Cre mice expressing mCherry control virus. One-way ANOVA, **P < 0.01 and ***P < 0.001; *n* = 4 animals per group. ns, not significant. (**G**) Food intake (30 min) in fasted Htr2a-Cre mice expressing hM4D(Gi) virus. One-way ANOVA, *P < 0.05 and **P < 0.01; *n* = 4 animals per group. (**H**) Food intake (30 min) in satiated Htr2a-Cre mice expressing hM3Dq virus. One-way ANOVA, *P < 0.05, **P < 0.01, and ****P < 0.0001; *n* = 8 animals per group. (**I**) Cannula implantation to infuse (250 nl) saline, ghrelin (1 μg), naloxone (50 μg), or JMV2959 (5 μg) into the CeA. (**J**) Representative image of CeA after infusion of the green fluorescent dye Lucifer yellow. Scale bar, 250 μm. (**K**) Food intake (1 hour) after the infusions in CeA described in (J). The “*” symbol indicates comparison with Saline + Saline, while the “#” symbol indicates comparisons with Saline + Ghrelin. One-way ANOVA, ****P < 0.0001, ^###^*P* < 0.001, and ^####^*P* < 0.0001; *n* = 8 animals per group.

To evaluate the role of CeA^Htr2a^ neurons in food intake induced by exogenous ghrelin, we stereotactically injected pAAV-hSyn-DIO-hM4D(Gi)-mCherry, pAAV-hSyn-DIO-hM3Dq-mCherry, or pAAV-hSyn-DIO-mCherry (control) into the CeA of Htr2a-Cre animals to express the inhibitory or excitatory designer receptor exclusively activated by designer drugs (DREADD) in CeA^Htr2a^ cells (Fig. 4, A and B). Infusion of the ligand clozapine *N*-oxide (CNO) efficiently inhibited CeA^Htr2a^ neurons expressing hM4D(Gi) (Fig. 4C and fig. S7, A and B) and excited the neurons expressing hM3Dq (Fig. 4D and fig. S7C). Since CeA^Htr2a^ neurons have the strongest impact on feeding when consumption is driven by palatability rather than homeostatic need (*6*), we evaluated food intake of satiated animals (fig. S7, D to G). Already 30 min after ghrelin intraperitoneal injections, the animals had increased their food intake in comparison with mice that received saline intraperitoneal injections (Fig. 4, E, F, and H). Chemoinhibition of CeA^Htr2a^ neurons completely abolished the ghrelin-induced food intake without altering the basal low food intake of satiated mice (Fig. 4E). Injections of the control pAAV-hSyn-DIO-mCherry virus followed by CNO application did not alter food intake induced by ghrelin injections (Fig. 4F and fig. S7E).

Fasting is known to increase endogenous ghrelin levels, but also engages the hypothalamic hunger neurons (*21*, *47*). As expected under these conditions, the activities of CeA^Htr2a^ neurons were not required for food intake (Fig. 4G and fig. S7F). These results indicate that the activities of CeA^Htr2a^ neurons are required for enhanced food intake induced by exogenous ghrelin.

Next, we asked if chemoactivation of CeA^Htr2a^ neurons by excitatory DREADDs (pAA-hSyn-DIO-hM3Dq-mCherry) could have an additive effect on food intake when paired with systemic ghrelin injections (Fig. 4H). Food intake after 30 min increased after systemic ghrelin injections and, to a lesser extent, after chemoactivation of CeA^Htr2a^ neurons as was previously shown (*6*). Both manipulations combined resulted in a further increase in food intake (Fig. 4H). Together, these results indicate that the activities of CeA^Htr2a^ neurons influence in a bidirectional manner food intake induced by exogenous ghrelin.

### Ghrelin action on CeA neurons is direct and depends on opioid and ghrelin receptors

Ghrelin-responsive neurons have been described in several brain regions including hippocampus and hypothalamus, and the exact role of the CeA in ghrelin-induced food intake is unclear. We therefore asked if direct infusion of ghrelin into the CeA could have an orexigenic effect. To deliver ghrelin and receptor antagonists directly into the CeA, we implanted a cannula stereotactically in CeA and studied the effects on food intake of exogenous ghrelin in the presence or absence of the growth hormone secretagogue receptor (GSHR) antagonist JMV2959 (Fig. 4I). After the behavior, we evaluated the correct implantation of the cannula using the green fluorescent dye Lucifer yellow, showing the position of drug delivery (Fig. 4J). Direct infusion of ghrelin into the CeA of fed animals had a similar effect on food intake as systemic delivery (Fig. 4K), suggesting that at least part of the ghrelin effects was mediated via direct actions on the CeA. Moreover, JMV2959 blocked the increase in food intake induced by ghrelin, suggesting a requirement of GSHR receptors in meditating ghrelin action (Fig. 4K).

CeA neurons express not only ghrelin GHSR receptors (fig. S7, I to L) but also opioid receptors (*48*–*50*), and several studies suggested that ghrelin signals via GHSR and opioid receptors (*24*, *26*, *51*). In agreement, we found that systemic administration of the opioid receptor antagonist naloxone reduced food intake after systemic ghrelin injection (fig. S7M) and direct infusion of ghrelin into the CeA (Fig. 4K). In addition, whole-cell current-clamp recordings showed that ghrelin depolarized CeA^Htr2a^ neurons (fig. S7, N and O) and that naloxone blocked this effect (fig. S7, P and Q).

### Fasting and ghrelin activate CeA→PBN projectors and inhibit PBN neurons

A large number of efferent projections from the CeA to the PBN come from CeA^Htr2a^ neurons, and photoactivation of these projections enhanced food consumption (*6*). In a first approach, we retrogradely labeled all CeA→PBN projectors by injecting retrobeads in PBN (fig. S8A) and recorded EPSCs in brain slices before and after perfusion of ghrelin (fig. S8B). We found that the EPSC frequencies, but not EPSC amplitudes, increased in PBN-projecting neurons, but not in nearby unlabeled CeA neurons (fig. S8, C and D). These results suggest that ghrelin induces the release of glutamate specifically from presynaptic inputs to PBN-projecting cells, similar to the results observed with CeA^Htr2a^ cells (Fig. 3H).

**Fig. 5.**
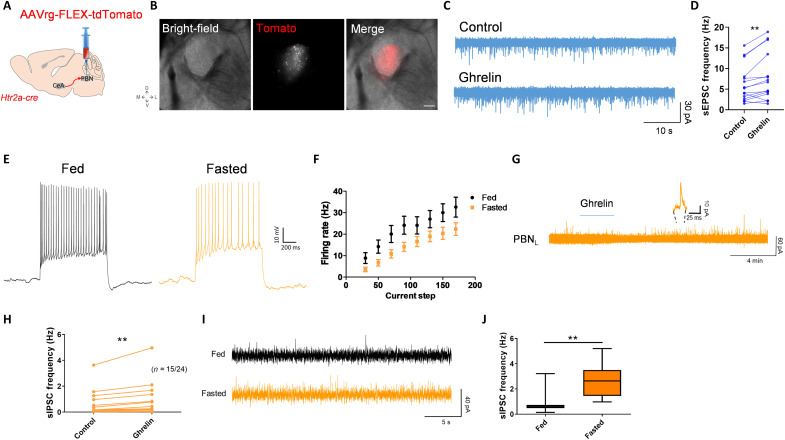
Serotonin receptor 2a (Htr2a)–expressing neurons (CeA^Htr2a^)→parabrachial nucleus (PBN) projectors increase activity after ghrelin perfusion resulting in PBN inhibition. (**A** and **B**) AAVrg-FLEX-tdTomato with a retrograde serotype (red) was stereotactically injected into PBN of Htr2a-Cre mice. CeA^Htr2a^→PBN projectors express tomato as shown in the representative images (B). Scale bar, 250 μm. (**C**) Representative spontaneous excitatory postsynaptic current (sEPSC) before (control) and after 3 min of ghrelin perfusion (ghrelin) in CeA^Htr2a^→PBN neurons. (**D**) sEPSC frequency from (C). *t* test, **P < 0.01; *n* = 15 neurons. (**E**) Whole-cell current-clamp recordings of PBN neurons from fed and fasted (20 hours) animals. (**F**) Firing rates (in hertz) after injecting different current steps in PBN neurons of fed (*n* = 20) and fasted (*n* = 23) animals. (**G**) Representative spontaneous inhibitory postsynaptic current (sIPSC) recording from PBN neurons, showing an increase in the frequency of inhibitory neurotransmission after the application of ghrelin (3 min, 100 nM). (**H**) Fifteen neurons of the 24 recorded in (G) increased sIPSC frequency and normalized ratio. Paired *t* test, **P < 0.01; *n* = 15. (**I**) Representative sIPSC recordings in PBN from fed and fasted (20 hours) animals. (**J**) Quantification of sIPSC frequency from fed (*n* = 11 cells) and fasted (*n* = 10 cells) animals. *t* test, **P < 0.01.

To specifically label CeA^Htr2a^ neurons projecting to PBN, we injected a Cre-dependent retrograde virus (AAVrg-FLEX-tdTomato) into PBN of Htr2a-Cre mice (Fig. 5A). CeA^Htr2a^ neurons expressing tdTomato (Fig. 5B) showed an increase in the frequency of sEPSC after perfusion of ghrelin (Fig. 5, C and D), similar to what was observed with retrobeads. If the PBN was under inhibitory control of CeA^Htr2a^ neurons, PBN neurons should get inhibited by the release of GABA. We found that the frequencies of action potentials in PBN neurons decreased after fasting (Fig. 5E), showing reduced firing rates after the injection of different current steps (Fig. 5F). Ghrelin perfusion in brain slices showed an increase in IPSC frequencies in the majority of PBN neurons (Fig. 5, G and H). Fasting produced a similar effect in the inhibition of PBN neurons than ghrelin (Fig. 5I), increasing the IPSC frequencies in fasted versus fed animals (Fig. 5J). These results suggest that CeA^Htr2a^→PBN projectors are activated by fasting and exogenous ghrelin, leading to inhibition of their target PBN neurons. Hence, CeA^Htr2a^→PBN projectors may act in parallel to GABAergic Agouti-related protein (AGRP) neurons of the arcuate region of the hypothalamus that also project to the PBN and promote feeding by inhibiting their target PBN neurons (*52*).

### Fasting and ghrelin activate CeA→PBN projectors to enhance food intake

**Fig. 6.**
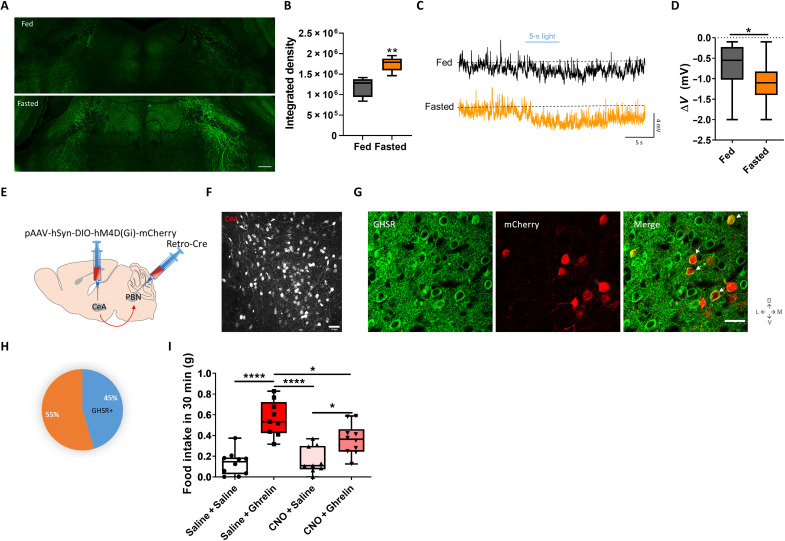
Ghrelin activates central amygdala (CeA) neurons inhibiting parabrachial nucleus (PBN) and inducing feeding. (**A**) Enhanced yellow fluorescent protein (EYFP) immunostainings in PBN from fed versus fasted animals injected with FosCh virus in CeA. Scale bar, 250 μm. (**B**) EYFP integrated density for the fluorescence in PBN from (A). *t* test, **P < 0.01; *n* = 5 sections from *n* = 3 animals per group. (**C**) Whole-cell current-clamp recordings with blue light stimulation (5 s) of PBN neurons from fed and fasted animals injected with FosCh virus in CeA. (**D**) Differences in membrane potentials (Δ*V*) of fed and fasted animals from (E). Paired *t* test, *P < 0.05; *n* = 16 cells. (**E**) Delivery of a retro-cre virus into PBN and of pAAV-hSyn-DIO-hM4D(Gi)-mCherry into CeA. (**F**) CeA→PBN projectors express hM4D(Gi)-mCherry (red). Scale bar, 50 μm. (**G**) Immunostainings showing the expression of growth hormone secretagogue receptor (GHSR) (green) and mCherry (red). Scale bar, 50 μm. (**H**) Percentage of mCherry-positive cells (CeA→PBN projectors) that express GHSR. (**I**) Chemogenetic inhibition of CeA→PBN projectors expressing hM4D(Gi) partially blocked the ghrelin-induced food intake (30 min) in satiated mice, compared to nonchemogenetically inhibited mice. One-way analysis of variance (ANOVA), *P < 0.05 and ****P < 0.0001; *n* = 8 mice per group.

To explore the role of the CeA→PBN projection in ghrelin-induced food uptake, we injected FosCh into CeA (as shown in Fig. 2A), fasted the animals to induce expression of FosCh, and followed the axonal long-range projections to different regions of the brain. We found increased expression of FosCh in the axons arriving in PBN, showing that fasting activated CeA→PBN projectors (Fig. 6, A and B). We recorded the membrane potentials of PBN neurons while inducing the release of GABA from CeA neurons with blue light. We found that PBN neurons hyperpolarized more in fasted than fed animals, most likely because the numbers of FosCh-expressing CeA neurons had increased during fasting (Fig. 6, C and D).

Next, we evaluated the requirement of CeA→PBN projectors in ghrelin-induced feeding behavior. We injected retro-cre virus bilaterally into PBN; thus, neurons in CeA projecting to PBN expressed the recombinase Cre. Then, we injected Cre-dependent inhibitory DREADDs in CeA (Fig. 6E). Neurons in CeA that projected to PBN expressed mCherry (Fig. 6F), and 45% of them expressed the ghrelin receptor GHSR (Fig. 6, G and H). We measured the cumulative food intake for 2 hours after intraperitoneal injections of different combinations of saline, CNO, and ghrelin (fig. S7H). The results showed that the ghrelin-induced food intake could be partially blocked by chemoinhibition of CeA→PBN projectors (Fig. 6I). Together, these results indicate that fasting activates CeA→PBN projectors, thereby inhibiting their target PBN neurons. They further show that the activities of CeA→PBN projectors strongly contribute to ghrelin-induced food intake.

### Transcriptional plasticity in specific CeA clusters

**Fig. 7.**
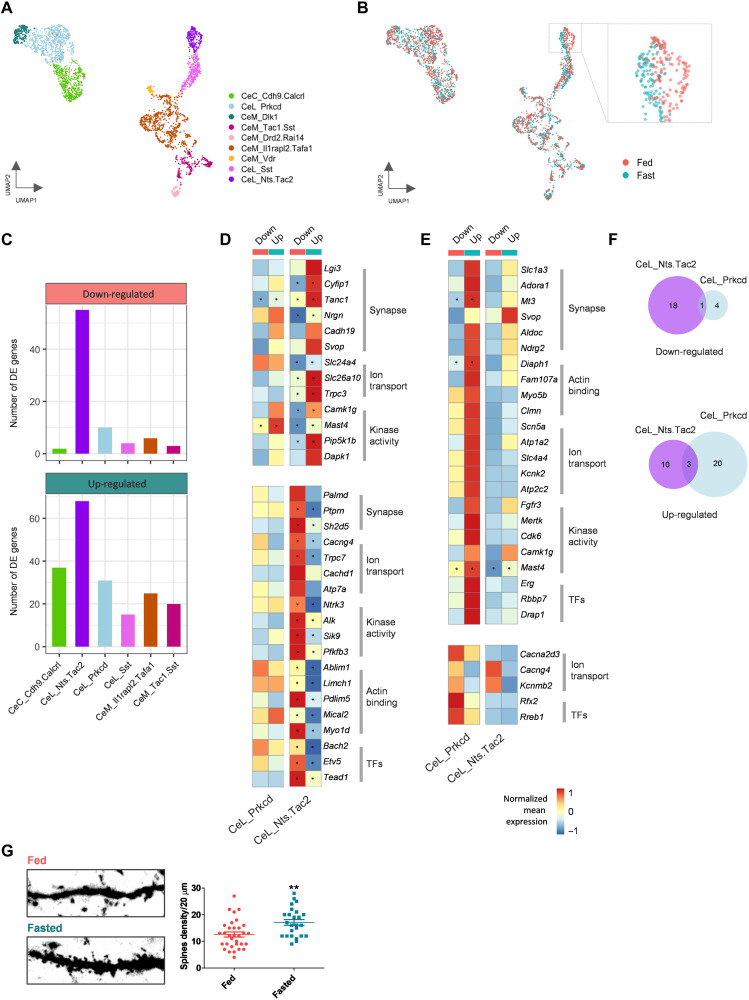
Identification of brain state–related differential gene expression. (**A** and **B**) Uniform Manifold Approximation and Projection (UMAP) representations of central amygdala (CeA) neurons, colored by cell clusters (A) or brain state groups (B). Inset in (B) indicates the CeL_NTS/Tac2 cell cluster. (**C**) Numbers of differentially expressed (DE) genes down-regulated or up-regulated in fasted groups found by the pseudo-bulk method edgeR-LRT; the threshold for determining the DE genes was adjusted *P* < 0.05 (Benjamini-Hochberg). (**D** and **E**) Heatmap of average gene expression across two cell types, the CeL_Prkcd and CeL_Nts.Tac2, in comparison of each of the two brain state groups. Genes shown were up-regulated (top) and down-regulated (bottom) in fasted groups in the CeL_Nts.Tac2 (D) and CeL_Prkcd (E) cell types (*P* < 0.01). Genes were listed from categories with Gene Ontology (GO) keywords (as indicated) that associated with neuronal activities. Asterisks indicate the genes revealed by adjusted *P* < 0.05 as in (C). Color bar: Log_2_-scaled mean expression. Value was centralized and row-scaled throughout each of the condition across both cell types. (**F**) Venn diagrams showing the numbers of genes listed in (D) and (E), separated by enriched in fed (top) or fasted (bottom) conditions. Note that there is very little overlap between the DE genes in CeL_Nts.Tac2 and CeL_Prkcd clusters. (**G**) Htr2a-Cre animals were injected with an AAV-DIO-GFP virus. Representative images of CeA^Htr2a^ dendritic spines (20 μm length) in fed versus fasted (20 hours) mice (left) and the corresponding quantification (right) are shown. *t* test, **P < 0.01; *n* = ~30 images from four amygdalas in each group.

To investigate whether appetitive and aversive CeA populations show transcriptional changes in response to fasting, we compared the transcriptomes of CeA cell clusters from both normal (fed) and food-deprived (fasted) mouse brains. In addition to the 3325 single nuclei from fed mice (Fig. 1), we added 3094 single nuclei from the CeA of mice that had been fasted. The nuclei from different batches mixed well at a low-dimensional embedding, indicating that the biological replicates did not exhibit any notable batch effects (fig. S9, A and B). We annotated the merged dataset based on previously identified transcriptomic markers (Fig. 1 and fig. S9C). Among the 16 clusters, astrocytes and oligodendrocytes showed the strongest differences between the fed and fasted groups, including changes in gene expression that are related to cytoplasmic translation (ribosomal subunits and translation elongation factors) and oxidoreductase activity (respiratory chain complex genes) (fig. S9, D and E). To dissect more nuanced transcriptional changes in response to fasting, we iteratively clustered GABAergic neurons of the CeA (see Materials and Methods; Fig. 7A). The CeL^NTS/Tac2^ cluster on the UMAP embedding exhibited a strong cell state change in response to fasting, suggesting that gene expression was strongly altered in this cell type (Fig. 7B). To reveal cell type–specific changes between fasted animals compared to ad libitum fed animals, we performed a pseudo-bulk differential gene expression analysis [edgeR-LRT; (*53*)] and compared the number of genes with a false discovery rate (FDR)–adjusted *P* value lower than 0.05. All clusters showed a moderate increase in the number of DE genes in the fasted state. However, the cluster CeL^NTS/Tac2^ displayed by far the largest number of DE genes in both up- and down-regulated genes, followed by CeL^PKCδ^ (Prkcd) (Fig. 7C). A Gene Ontology (GO) enrichment analysis of the DE genes in the CeL^NTS/Tac2^ and CeL^PKCδ^ populations revealed GO terms related to neuronal activity (synapse, ion transport, kinase activity, and actin binding) and transcription factors (Fig. 7, D and E). The results showed a high degree of specificity, with only a limited number of genes shared between the two clusters (Fig. 7F).

During fasting, several genes related to an increase in synaptic plasticity and dendritic spine formation were up-regulated in the CeL^NTS/Tac2^ cluster. They include *DAPK1* (*death associated protein kinase 1*) that phosphorylates glutamate ionotropic receptor *N*-methyl-d-aspartate (NMDA) type subunit 2B, increasing Ca^2+^ influx through NMDA receptor channels (*54*), *CYFIP1* (*cytoplasmic FMR1 interacting protein 1*) that ensures proper dendritic spine formation inducing protein translation and actin polymerization (*55*), and *TANC1* (*tetratricopeptide repeat, ankyrin repeat and coiled-coil containing 1*), a PSD-95–interacting synaptic protein that increases the density of dendritic spines and excitatory synapses (*56*). Conversely, genes that decrease dendritic spine formation were down-regulated in CeL^NTS/Tac2^ cells while fasting. They include *PDLIM5* (*PDZ and LIM domain 5*) that functions in shrinkage of dendritic spines, restraining postsynaptic growth of excitatory synapses (*57*), and *MICAL2* (*microtubule associated monooxygenase, calponin and LIM domain containing 2*) that regulates the disassembly of branched actin networks (*58*).

CeA^Htr2a^ neurons consist of three presumed appetitive clusters (CeL^NTS/Tac2^, CeL^Sst^, and CeM^IL1Rapl2^), only one of which (CeL^NTS/Tac2^) showed cell state changes in response to fasting. Hence, the response of CeA^Htr2a^ neurons to fasting was heterogeneous, with some cells displaying a large number of differentially expressed (DE) genes and others not. Despite this heterogeneity, we investigated if fasting may cause structural plasticity in (a subset of) CeA^Htr2a^ neurons. We evaluated if fasting promoted dendritic spine formation in CeA^Htr2a^ neurons. Imaging random stretches of dendrites expressing YFP revealed a ~26% increase in spine density in CeA^Htr2a^ neurons in fasted versus fed mice (Fig. 7G). Together, these results suggest that a subset of CeA neurons responded to fasting with dynamic gene expression changes and increases in the number of dendritic spines.

## DISCUSSION

In this report, we have described a transcriptomic taxonomy of GABAergic cell clusters in the adult CeA. We identified a total of nine cell clusters, of which six had previously been functionally analyzed. On the basis of prior literature (*7*), two cell clusters (CeC^Calcrl^ and CeL^PKCδ^) were mostly associated with aversive behaviors, whereas four clusters (CeL^NTS/Tac2^, CeL^Sst^, CeM^Il1Rapl2^, and CeM^Tac1/Sst^) were mostly, but not exclusively, associated with appetitive behaviors. CeA^Htr2a^ neurons, previously shown to promote feeding (*6*), were found to comprise three appetitive (and one uncharacterized) cell clusters. We went on to explore how appetitive CeA neurons are physiologically activated and used Htr2a-Cre animals for functional analysis. c-Fos stainings, electrophysiological slice recordings, and in vivo calcium imaging revealed that appetitive CeA^Htr2a^ neurons are activated by the presence of food and in animals subjected to fasting. Fasting is known to induce the expression of endogenous ghrelin (*23*, *59*), and we confirmed that enhanced food consumption after fasting requires endogenous ghrelin signaling. Here, we found that ghrelin administration activated CeA^Htr2a^ neurons in vitro and in vivo. Moreover, the actions of ghrelin seem to be directly targeting CeA^Htr2a^ neurons and their excitatory inputs, and the activity of CeA^Htr2a^ neurons is required for the orexigenic effects of ghrelin. Our results further suggest that appetitive CeA neurons responsive to fasting and ghrelin project to the PBN, resulting in an inhibition of target PBN neurons. Last, we found that a subset of presumed appetitive CeA neurons showed dynamic and bidirectional gene expression changes in response to fasting suggestive of plasticity at synaptic sites. These findings enhance our understanding of cell type diversity in CeA and the role of CeA cell populations in the hormonal regulation of food consumption.

The four appetitive cell clusters identified by our snRNA-seq analysis largely matched previous work using single molecular markers, although some questions remain unanswered. In the CeL, we found two clusters marked by expression of *Sst*: the CeL^Sst^ cluster, which contains the largest and most highly expressing population of Sst-positive neurons, and the CeL^NTS/Tac2^ cluster, which contains low expressing Sst-positive cells. This is consistent with previous observations (*8*, *12*) and recent single-cell transcriptomics studies in the CeA (*60*, *61*). Optogenetic manipulation of these two clusters separately suggested that both drive appetitive behavior (*8*). Consistent with such a notion, NTS-positive neurons enriched in the CeL^NTS/Tac2^ cluster (Fig. 1H) were previously shown to promote consumption of palatable fluids (*17*). However, the entire population of Sst-positive neurons in CeL can also undergo synaptic potentiation during fear conditioning and facilitate the expression of conditioned freezing behavior (*10*, *14*, *16*). Likewise, corticotropin-releasing factor (CRF or CRH) neurons that are largely contained in the appetitive CeL^NTS/Tac2^ cluster and in a somewhat sparser CeM population (*8*, *12*) were shown to be important for fear learning and active fear responses (*43*, *62*). It therefore remains an open question whether Sst- and CRH-positive neurons are more heterogeneous than suggested from their transcriptomes and consist of appetitive and aversive neurons, or, alternatively, whether each neuron can be involved in a broader range of approaches and defensive behaviors. Future studies will have to explore the specificity or generality of distinct CeA populations (*63*).

In the CeM, we found two additional cell clusters: the large IlRapl2 and the smaller Tac1/Sst cluster. The CeM^IlRapl2^ cluster, which cannot easily be addressed with a single marker (*61*), contains the CeM subsets of the CeA^Pnoc^ and CeA^Htr2a^ neurons (Fig. 1, I to L). Since both neuron populations were previously shown to promote food uptake and reward behavior (*5*, *6*), the results suggest that this large CeM cluster is mostly involved in appetitive behavior. Future work involving intersectional genetic approaches (*64*) should further explore the relative contributions of the CeL and CeM subsets of these populations. The CeM^Tac1/Sst^ cluster remains largely unexplored, except for the previous observation that optogenetic activation of the CeM subset of Sst cells promoted rewarding behavior (*8*).

Our transcriptomic mapping separated PKCδ neurons into two clusters: one in CeL (PKCδ) and one in CeC (Calcrl). This result is consistent with recent transcriptomics studies (*60*, *61*) and previous gene expression analysis (*8*). Both CeL^PKCδ^ and CeC^Calcrl^ neurons were previously shown to be involved in defensive behaviors (*7*, *37*, *40*, *65*). More recently, PKCδ-positive neurons were also implicated in appetitive/affective behavior (*38*, *39*). Future work is needed to sort out these seemingly inconsistent observations. Moreover, our discovery of three previously uncharacterized CeM clusters (CeM^Vdr^, CeM^Drd2/Rai14^, and CeM^Dlk1^) could be a starting point of a comprehensive functional analysis of this understudied subregion of the CeA.

This study also revealed an interesting hormonal regulation of appetitive CeA neurons by ghrelin. Our electrophysiological recordings suggest that ghrelin can act directly on CeA neurons projecting to the PBN (since ghrelin increases inhibition of PBN neurons) and on their excitatory inputs to increase the excitability of the CeA neurons. This observation adds the CeA to the list of extrahypothalamic ghrelin targets, including VTA, NAc, hippocampus, and the BLA (*20*–*22*). Mechanistically, ghrelin may be acting through GHSR and/or opioid pathways, possibly by heteromerization of the receptors or the downstream signaling pathways (*24*–*26*, *51*, *66*). The activation of opioid receptors in CeA has been shown to increase food intake (*67*), and ghrelin has antinociceptive effects on irritable bowel syndrome depending on the opioid system (*66*). Therefore, our observation that ghrelin-induced feeding is strongly inhibited by the opioid receptor inhibitor naloxone could be an interesting topic for further research.

In vivo calcium imaging revealed multiple ways that appetitive CeA^Htr2a^ neurons become activated. We found that in satiated mice, the presence of food increased the activities of the majority of CeA^Htr2a^ neurons, although food consumption was minor. In the satiated state in the absence of food, exogenous ghrelin increased CeA^Htr2a^ neuronal activities, and in the presence of food, CeA^Htr2a^ neuronal activities strongly correlated with feeding. This supports the notion that this subpopulation of cells is relevant for the modulation of food consumption through a positive-valence mechanism (*6*). The results further suggest that ghrelin predisposes those cells for the process of eating. As previously shown in humans, ghrelin may favor food consumption by enhancing the rewarding and incentive responses to food-related cues (*22*). Fasting recruited more cells to the active ensembles similarly to ghrelin injections, suggesting that endogenous ghrelin released while fasting also produces a similar effect. The activity of CeA^Htr2a^ neurons was required for the orexigenic effects of ghrelin, only when administered to satiated, not hungry mice, consistent with the idea that this process drives the sensory perception or pleasure of feeding (*1*, *6*, *68*). Together, these findings suggest a model in which palatable food engages CeA^Htr2a^ neurons. The hungrier the animals are, the more palatable the food becomes. As ghrelin levels rise during fasting, CeA^Htr2a^ neurons are activated and contribute to the process of palatability-guided feeding.

At the microcircuit level, our results revealed that fasting and exogenous ghrelin activated CeA neurons that project to the PBN, resulting in hyperpolarization and inhibition of their downstream PBN targets. Chemoinhibition of the CeA→PBN projectors, which consist mainly of CeA^Htr2a^ neurons (*6*), partially blocked ghrelin-induced food intake. The identity of PBN neurons engaged by CeA^Htr2a^ neurons remains to be elucidated.

We found that fasting induced gene expression changes in specific CeA neuron clusters, most pronounced and bidirectional in the CeL^NTS/Tac2^ cluster. These results are reminiscent of the hypothalamic feeding circuit, where appetitive AGRP neurons undergo more extensive gene expression changes after fasting than appetite-suppressing Pro-opiomelanocortin (POMC) neurons (*69*). The types of differentially expressed genes (DEG) were similar to appetitive CeA neurons, associated with alterations in synaptic proteins, kinases, and ion channels (*69*). For example, we found that *anaplastic lymphoma kinase* (*Alk*) expression was down-regulated in CeL^NTS/Tac2^ neurons in fasted mice (Fig. 7D), a gene whose genetic deletion was previously shown to result in thin animals with resistance to diet- and leptin mutation–induced obesity, acting mainly in the hypothalamus (*70*). The up- and down-regulation of genes controlling synaptic plasticity in CeL^NTS/Tac2^ cells while fasting (up: *DAPK1*, *CYFIP1*, *TANC1*; down: *PDLIM5*, *MICAL2*) is consistent with a model in which appetitive neurons modify their structure to receive more excitatory synapses, as observed in electrophysiological recordings. Fasting also increased electrical activity and synaptic plasticity in AGRP neurons (*71*, *72*), an observation that is paralleled in appetitive CeA neurons. Ghrelin is a likely mediator of some of these gene expression changes, as shown for AGRP neurons (*73*). Ghrelin also induces spine growth and synaptic plasticity in areas like the hippocampus or hypothalamus (*47*, *74*, *75*). Our results also showed an increase in spine density in CeA^Htr2a^ neurons while fasting, which correlated with the changes in DE genes in the CeL^NTS/Tac2^ cluster.

In conclusion, our transcriptomic taxonomy illustrates the diversification of CeA neurons and shows how the activity of appetitive neurons in the CeA is regulated by interoceptive cues (fasting and ghrelin) and the presence of food. Appetitive CeA^Htr2a^ neurons are essential for the orexigenic effects of ghrelin. They project to the PBN causing inhibition of target PBN neurons. Our findings reveal a previously unidentified role of the CeA in the regulation of feeding behavior by the ghrelin system. In addition, our results lay the groundwork for further investigations of how specific CeA neuron populations interact with the hedonic and homeostatic feeding circuits, and the interactions among emotional states, food intake, and reward. Last, the malfunction of CeA neurons and microcircuits may underlie pathological eating behavior.

## MATERIALS AND METHODS

### Animals

Experiments were always performed using adult mice (>8 weeks). The wild-type animals were from the C57BL/6NRj strain (Janvier Labs, www.janvier-labs.com). The Htr2a-Cre bacterial artificial chromosome (BAC) transgenic line [stock Tg(Htr2a-Cre)KM208Gsat/Mmucd] and Prkcd-Cre [Tg(Prkcd-glc-1/CFP-Cre)EH124Gsat] BAC mice were imported from the Mutant Mouse Regional Resource Center (www.mmrrc.org/). Td-Tomato Rosa26R mouse lines were as described previously (*76*), using the line Ai9lsl-tdTomato [B6.Cg-Gt(ROSA)26SorTM9.CAG-tdTomato/Hze/J] (*77*, *78*). Transgenic mice were backcrossed with a C57BL/6N background. Animals used for opto- and chemogenetic manipulations were handled and singly housed on a 12-hour inverted light cycle for at least 3 days before the experiments.

Mice were given ad libitum food access except during food deprivation for feeding experiments. All feeding behavior assays were conducted at a consistent time during the dark period (2 p.m.to 7 p.m.). Both male and female mice were used, and all the experiments were performed following regulations from the government of Upper Bavaria. The animal study was reviewed and approved by the Regierung von Oberbayern under the license 55.2-2532.Vet_02-20-49.

### Viral constructs

The following AAV (adeno-associated virus) viruses were produced at the Gene Therapy Center Vector Core at the University of North Carolina Chapel Hill: AAV-cFos-hChR2(H134R)-EYFP-Pest-no-WPRE, AAV-hSyn-EYFP, AAV8-hSyn-DIO-hM3D(Gq)-mCherry, and pAAV-EF1a-DIO-mCherry (UNC Vector Core, USA). The AAV5-Syn.Flex.GCaMP6s virus was obtained from the Penn Vector Core (USA). pAAV-hSyn-DIO-hM4D(Gi)-mCherry, pAAV-hSyn-DIO-mCherry, and pAAV-FLEX-tdTomato (produced with a retrograde serotype AAVrg) were obtained from Addgene (USA).

### Tissue dissection for snRNA-seq

To minimize potential batch effects, each experiment or dataset consisted of CeA tissue from one male and one female brain, collected, and processed simultaneously and in parallel. The mice were deeply anesthetized using an intraperitoneal injection of ketamine (200 mg/kg) and xylazine (40 mg/kg). They were then perfused with 10 ml of ice-cold “Cutting Buffer” consisting of sucrose-Hepes containing 110 mM NaCl, 2.5 mM KCl, 10 mM Hepes, 7.5 mM MgCl_2_, 25 mM glucose, and 75 mM sucrose (approximately 350 mOsm/kg) (pH 7.4) (*79*). All solutions and reagents were kept on ice, unless specified otherwise. The brain was removed and sliced into 300-μm sections using a vibratome (Leica VT1000S, Germany) in the cutting buffer. The slices were transferred into a “Dissociation Buffer” containing 82 mM Na_2_SO_4_, 30 mM K_2_SO_4_, 10 mM Hepes, 10 mM glucose, and 5 mM MgCl_2_ (pH 7.4). The CeA was microdissected under a microscope (Olympus SZX10).

### Single-nuclei isolation and library preparation

The protocol for single-nuclei isolation was adapted from a previous study (*80*). Briefly, collected tissue was transferred into 600 μl of homogenization buffer containing 320 mM sucrose, 5 mM CaCl_2_, 3 mM Mg(CH_3_COO)_2_, 10 mM tris-HCl (pH 7.8), 0.1 mM EDTA (pH 8.0), 0.1% NP-40 (70% in H_2_O; NP40S, Sigma-Aldrich), 1 mM β-mercaptoethanol, and SUPERase ribonuclease inhibitor (0.4 U/μl; AM2694, Invitrogen). The tissue was homogenized in a 1-ml Wheaton Dounce, filtered through a 20-μm cell strainer (Miltenyi Biotec), and mixed with an equal volume of working solution [50% OptiPrep density gradient medium (Sigma-Aldrich), 5 mM CaCl_2_, 3 mM Mg(CH_3_COO)_2_, 10 mM tris-HCl (pH 7.8), 0.1 mM EDTA (pH 8.0), and 1 mM β-mercaptoethanol]. The resulting solution was transferred into a 2-ml centrifuge tube containing a 29% OptiPrep density gradient solution [134 mM sucrose, 5 mM CaCl_2_, 3 mM Mg(CH_3_COO)_2_, 10 mM tris-HCl (pH 7.8), 0.1 mM EDTA (pH 8.0), 1 mM β-mercaptoethanol, 0.04% NP-40, and SUPERase inhibitor (0.17 U/μl)]. A 35% density solution [96 mM sucrose, 5 mM CaCl_2_, 3 mM Mg(CH_3_COO)_2_, 10 mM tris-HCl (pH 7.8), 0.1 mM EDTA (pH 8.0), 1 mM β-mercaptoethanol, 0.03% NP-40, and SUPERase inhibitor (0.12 U/μl)] was slowly added below the 29% density, and the nuclei were separated by ultracentrifugation using an SH 3000 rotor (20 min, 3000*g*, 4°C). Three hundred microliters of nuclei was collected from the 29%/35% interphase and washed once with 2 ml of resuspension solution [0.3% bovine serum albumin (BSA) and SUPERase (0.2 U/μl) in phosphate-buffered saline (PBS)]. The nuclei were centrifuged (300*g*) for 5 min and resuspended in ~30 μl of resuspension solution.

The nuclei were stained with 4′,6-diamidino-2-phenylindole (DAPI) and counted. Around 100 to 200 nuclei/μl suspension was obtained and immediately added to a microfluidic platform (10x Genomics). Nanoliter-scale Gel Beads-in-emulsion (GEMs) generation, barcoding, cDNA amplification, and library preparation were done using the Chromium Next GEM Single Cell 3′ Reagent Kits v3.1 according to the manufacturer’s protocol.

### Sequence alignment, pre-processing, and clustering

Raw reads were obtained from NovaSeq 6000 (Helmholtz Zentrum München, Germany) and converted to FASTQ files, followed by sequencing alignment using the Cell Ranger (V5.0.0, 10x Genomics) pipeline. The mouse reference genome GRCm39 release 105 (http://ftp.ensembl.org/pub/release-105/fasta/mus_musculus/dna/) and annotation files (http://ftp.ensembl.org/pub/release-105/gtf/mus_musculus/) were modified to include the sequence of Cre recombinase (www.ncbi.nlm.nih.gov/nuccore/NC_005856.1?report=fasta&from=436&to=1467) and the open reading frame of mCherry from the viral construct pAAV-EF1a-DIO-mCherry (www.addgene.org/50462/sequences/). Sequencing reads were aligned with the reference genome, and gene–by–cell count matrices were generated using “cell ranger count” command with default parameters, including the “--include-introns = true” argument.

Seurat V4.0.1 was used for subsequent data filtering, normalization, clustering, and visualization. The percentage of mitochondrial genes and the number of unique molecular identifier (UMI) counts per nucleus were calculated, as well as the number of genes detected per nucleus. Nuclei with extreme values of these quality metrics were filtered out on the basis of the distribution of each dataset. Generally, nuclei with a number of genes detected per nucleus between 500 and 8000 and a percentage of mitochondrial genes less than 0.8% were kept. The datasets were then normalized using the “SCTransform” function in Seurat with the regularized negative binomial regression method, using the percentage of mitochondrial genes as a confounding source of variance to be regressed out. As no global batch effect that separated the datasets was observed, they were merged, and the top 2000 HVGs were selected across all datasets. Three datasets from satiated mice were used as reference for CeA dataset, and five datasets from all experiments were used to compare fasted and fed conditions. PCA was performed on the scaled expression of the HVGs for each merged dataset, followed by graph-based clustering using the “FindNeighbors” and “FindClusters” functions, and UMAP visualization using the “RunUMAP” function. Coarse clusters were classified using the parameter “resolution = 0.5” for the first round of clustering (with all cell types). Gad1/2-positive inhibitory neurons were clustered iteratively with “resolution = 0.8” to obtain more refined clusters.

To determine the final number of CeA neuronal clusters, two factors were considered: (i) obtaining the minimum number of clusters that display a distinct distribution of cell types on low-dimensional embeddings, such as UMAP, and (ii) incorporating prior knowledge to annotate cell types increasing the cluster resolution.

### Cell type annotation

To annotate the CeA neuronal types, we first performed DE analysis for each cluster using the “FindAllMarkers” function with default parameters. The following criteria were used to select the representative markers for a cell type: (i) conventional markers previously reported; (ii) expression patterns of the top 20 DE genes, designating the one with higher specificity as its marker; (iii) two markers were used when only one was not enough; and (iv) genes with entries in the Allen Reference Atlas—Developing Mouse Brain (https://developingmouse.brain-map.org/). Only genes showing specific expression patterns in the CeA subdivisions (CeL, CeM, or CeC) were selected as markers. To name the clusters, the following nomenclature was used: sub-division_marker or sub-division_marker1.marker2.

To visualize the expression of marker genes, either the “DotPlot” or “FeaturePlot” function in Seurat was used. To depict the expression of appetitive cell type markers, such as *Pnoc* and *Htr2a-Cre*, a density plot was created on the basis of the kernel density estimation using the “Nebulosa” package (*81*). To annotate the CeA neuronal types in the merged datasets, which included both the fed and fasted groups, a Pearson correlation matrix was constructed between the previously annotated dataset and the clusters from the merged dataset, using the mean expression of the 2000 HVGs. The latter were named on the basis of the highest correlation between cluster pairs and were further analyzed on the basis of the expression patterns of previously identified markers for the nine CeA cell types. To visualize the marker gene expression, either the “DotPlot” or “FeaturePlot” function in Seurat was used.

### Construction of cell type taxonomy tree

To construct the cell type hierarchy, the average expression of the 2000 previously identified high variable genes was calculated for each cluster. A Pearson correlation matrix was then created to compare the expression of these genes between the cell types, and hierarchical clustering with the average linkage method was applied to infer the taxonomic tree structure.

### DE analysis

To compare the differential gene programs between fasted and fed conditions in astrocytes and oligodendrocytes, DE analysis was performed similarly as described above, with a stricter threshold condition. Genes with a log_2_-transformed average fold change (avg_log2FC) greater than 0.25 and a *P* value (obtained from a Wilcoxon rank sum test) less than 0.001 were considered DE.

For the DE analysis of CeA neuronal cell types, the pseudo-bulk–based method edgeR from the “Libra” package was used. A two-threshold strategy was used: Genes with an FDR-adjusted *P* value (corrected using the Benjamini-Hochberg method) less than 0.05 were considered DE, while genes with a *P* value directly calculated from the likelihood ratio test (edgeR-LRT) less than 0.01 were also included.

### GO enrichment

Up-regulated and down-regulated genes were separately analyzed for gene category enrichment using DAVID (Database for Annotation, Visualization, and Integrated Discovery; https://david.ncifcrf.gov/), a high-throughput and integrated data-mining web server (*82*). This tool was used to identify representative GO terms/keywords and enriched pathways.

### RNAscope experiments

Multicolor fluorescence in situ hybridization was performed on fixed frozen brain slices. Briefly, brain tissue was embedded in O.C.T. (Sakura Finetek, 4583) in cryomolds (Sakura Finetek, 4566) and fresh-frozen on dry ice. Brain tissue was cut on a cryostat (CM3050S, Leica) to obtain 20-μm sections, collected on slides (48311-703, VWR Microslides Superfrost Plus), and stored at −80°C. Hybridization was performed using the RNAscope Kit (ACDBio). Probes against Dlk1 (405971-C2), (439371), and Sst (404631) were applied at a 1:50 dilution to sections. Images were taken using a Leica SP8 confocal microscope and processed using ImageJ software [National Institutes of Health (NIH)].

### Food consumption measurements

Food restriction experiments were done only for a maximum of 20 hours to avoid major complications on mice’s health. The food was completely removed from the home cage, but keeping the water supply. After 20-hour fasting, one single pellet of food (previously weighted) was added to the cage, and the food consumption was measured. For feeding assays using satiated animals, different amounts of ghrelin (1 to 10 μg; #1465, Tocris), ghrelin receptor antagonist JMV2959 (250 μg; #345888, Calbiochem), or saline were intraperitoneally injected, and food intake was measured for the following 3 hours after injection. For calcium imaging experiments, we used dustless precision pellets 20 mg each (#F0071, Bio-Serv).

### Stereotactic surgeries

Mice were anesthetized using 1.5% isoflurane (Cp-pharma, Germany) (4% for induction) with the head fixed on a stereotactic frame (model 1900, Kopf Instruments). Body temperature was maintained at 37°C using a heating pad, and carprofen (Rimadyl, Zoetis, Germany) (5 mg/kg body weight) was subcutaneously administered as an analgesic.

Mice were injected bilaterally using glass pipettes (#708707, BLAUBRAND intraMARK) with 300 nl of the following viruses or beads: pAAV-hSyn-DIO-hM4D(Gi)-mCherry, AAV-cFos-hChR2(H134R)-EYFP-Pest, pAA-hSyn-DIO-hM3Dq-mCherry, pAAV-hSyn-DIO-mCherry, and Retrobeads (Lumafluor, USA). The stereotactic coordinates used for CeA were −1.22 mm anteroposterior (AP), ±2.8 mm medial-lateral (ML), and −4.72 mm dorso-ventral (DV); the stereotactic coordinates used for PBN were −5.3 mm AP, ±1.35 mm ML, and −3.9 mm DV. After the injection, the pipette remained in the brain for 4 min to prevent the spreading of the virus. The wound was closed with Vetbond (3M, USA), and the mice recovered in the home cage at a warm temperature.

Mice used in optogenetic experiments were bilaterally implanted with optic fibers (200-μm core, 0.22 numerical aperture, 1.25-mm ferrule) (Thorlabs, USA) above the CeA (−4.2 mm DV), and the implants were secured with self-curing acrylic resin (Paladur, Kulzer, Germany).

For the injection of substances directly into CeA, a thin guide cannula (26 gauge) was implanted bilaterally −4 mm DV, and a dummy cannula was inserted into CeA (−4.72 DV) using the stereotactic apparatus. The cannula was attached to the skull with Paladur, as described for optic fibers. On the day of surgery, and two subsequent days, carprofen [5 mg/kg, Zoetis (Rimadyl)] was also administered as an additional analgesic.

### Chemogenetic manipulations

For all the behavioral experiments involving chemogenetics, animals were habituated, handled, and injected intraperitoneally with saline for 3 days. For pharmacological treatments, mice stereotactically injected with DREADD or mCherry controls received 100 μl of intraperitoneal injection of CNO (0.4 or 1 mg/kg diluted in saline), ghrelin (10 μg, otherwise stated), or the equivalent volume of saline. The animals were injected using different time points (Fig. 4), allowing them to recover in their home cages for 30 min (for excitatory DREADD) or 1 hour (for inhibitory DREADD) before the second injection with ghrelin or saline. Food intake was measured for the following 3 hours. The experiments were performed randomly in duplicates on different days, with one resting day in between experiments.

### Optogenetic manipulations

Mice were bilaterally tied to optic-fiber patch cords (Doric Lenses or Thorlabs) connected to a 473-nm laser (CNI lasers; Cobolt) via a rotary joint (Doric Lenses) and mating sleeve (Thorlabs). Photostimulation was performed using 10-ms, 473-nm light pulses at 20 Hz and 10 mW. The laser was triggered, and pulses were controlled with Bonsai data-streaming software ref and Arduino microcontrollers (www.arduino.cc/).

### Drug infusions into the brain

The implanted animals were accustomed to the experimenter and the experimental setup. Briefly, mice were anesthetized with 1.5% isoflurane (Cp-pharma, Germany) and head-fixed on a stereotactic setup. Dummy cannula was removed and cleaned with 70% ethanol, and 0.25 μl of saline solution was injected using a Hamilton syringe with a 32-gauge blunt needle (7000 Series Neuros, Hamilton, USA) using −4.72 DV coordinates and kept inside for 1 min. Animals recovered in their home cage. For the experiments, two separate infusions of drugs were delivered into CeA with 10 min of difference to allow the antagonists to act against their receptors. The drugs used were ghrelin (1 μg; #1465, Tocris), JMV2959 (5 μg; #345888, Calbiochem), and naloxone (50 μg; ab120074, Abcam) in a volume of 0.25 μl with saline. After the two injections, the mice recovered in their home cages without food for 10 min, and then a preweighted food pellet was added into the cage, measuring the food intake for the following 2 hours.

### GRIN lens implantation and baseplate fixation

Three weeks after GCaMP6s virus injection, gradient index (GRIN) lenses were implanted in Htr2a-Cre mice as follows. The animals were located in the stereotactic setup under isoflurane anesthesia (see the “Stereotactic surgeries” section), and a small craniotomy was made above the CeA using the same coordinates as for the injection of the viral preparation. After removal of the debris, a blunted 23-gauge needle (0.7 mm in diameter) was slowly inserted into the brain to a depth of −4.6 mm from bregma. The needle was retracted, and a GRIN lens (ProView lense; diameter, 0.5 mm; length, ~8.4 mm, Inscopix) was implanted above the CeA. The skull was covered with a thin layer of histo glue (Histoacryl, Braun), the lens was then fixed to the skull using ultraviolet light curable glue (Loctite AA3491, Henkel), and the exposed skull was covered with dental acrylic (Paladur, Heraeus). The exposed top of the lens was protected by a covering of a silicone adhesive (Kwik-cast, World Precision Instruments). One to 2 months later, a baseplate (BPL-2; Inscopix) attached to the miniscope was positioned above the GRIN lens in the stereotactic setup under anesthesia. The focal plane was adjusted while lowering the concentration of the anesthetic gas until the GCaMP6s signal was observed. Last, mice were fully anesthetized again and the baseplate was fixed using C&B Metabond (Parkell). A baseplate cap (BCP-2, Inscopix) was left in place until imaging experiments.

### In vivo calcium imaging of freely moving mice

Experiments were conducted on freely moving mice expressing GCaMP6s virus in CeA^Htr2a^ cells. The head was fixed and the miniscope was secured in the baseplate holder. Mice were habituated to the miniscope 3 days before behavioral experiments. Settings were kept constant within the different experimental sessions. Imaging acquisition and behavior were synchronized using the data acquisition box of the nVoke Imaging System (Inscopix), triggered by the EthoVision XT 14 software (Noldus) through a TTL box (Noldus) connected to the USB-IO box from the EthoVision system (Noldus).

On the day of the experiments, mice were intraperitoneally injected with saline or ghrelin (1465, Tocris) acclimated in their cages for 10 min, and then compressed images were obtained at 20 Hz with Inscopix nVista HD V2 software. After 10 min of recording without food, the animals were exposed to food for an additional 10 min. The last experiment was also done fasting (20 hours) instead of injecting intraperitoneally ghrelin. For imaging data processing and analysis, we used the IDPS (Inscopix data processing software) version 1.8.0.

### Acute brain slice preparation and electrophysiological recordings

Mice were deeply anesthetized with isoflurane and decapitated. The brain was placed in an ice-cold cutting solution saturated with a mixture of 95% O_2_ and 5% CO_2_ containing 30 mM NaCl, 4.5 mM KCl, 1 mM MgCl_2_, 26 mM NaHCO_3_, 1.2 mM NaH_2_PO_4_, 10 mM glucose, and 194 mM sucrose. After slicing the brain at a thickness of 280 μm on a vibratome (Leica VT1000S, Germany), the slices were transferred into an artificial cerebrospinal fluid (aCSF) solution containing 124 mM NaCl, 4.5 mM KCl, 1 mM MgCl_2_, 26 mM NaHCO_3_, 1.2 mM NaH_2_PO_4_, 10 mM glucose, and 2 mM CaCl_2_ (310 to 320 mOsm), saturated with 95% O_2_/5% CO_2_ at ~32°C for 1 hour before being moved to room temperature (RT). Last, the brain slices were transferred to a recording chamber continuously perfused with aCSF solution saturated with 95% O_2_/5% CO_2_ at 30° to 32°C.

Whole-cell patch-clamp recordings were performed as previously described (*83*). Briefly, patch pipettes were prepared from filament-containing borosilicate micropipettes (World Precision Instruments) using a P-1000 micropipette puller (Sutter Instruments, Novato, CA), with a resistance of 5 to 7 megohms. The intracellular solution contained 130 mM potassium gluconate, 10 mM KCl, 2 mM MgCl_2_, 10 mM Hepes, 2 mM Na-ATP, 0.2 mM Na_2_GTP (pH7.35), and 290 mOsm. Slices were visualized with a fluorescence microscope equipped with IR-DIC optics (Olympus BX51). The holding potential for EPSC was set in −70 and 0 mV for IPSC. Data were obtained using a MultiClamp 700B amplifier, Digidata 1550 digitizer (Molecular Devices), and the software Clampex 10.3 (Molecular Devices, Sunnyvale, CA). Data were sampled at 10 kHz, filtered at 2 kHz, and analyzed with Clampfit (Molecular Devices).

For optogenetic studies, neurons were stimulated using a multi-LED (light-emitting diode) array system (CoolLED) connected to an Olympus BX51 microscope.

### Histology

Animals were anesthetized intraperitoneally with a mix of ketamine/xylazine (100 and 16 mg/kg, respectively) (Medistar and Serumwerk) and transcardially perfused with ice-cold PBS, followed by 4% (w/v) paraformaldehyde (PFA) (1004005, Merck) in PBS. Brains were postfixed at 4°C in 4% (w/v) PFA in PBS overnight, embedded in 4% (w/v) agarose (#01280, Biomol) in PBS, and sliced (50 to 100 μm) using a Vibratome (VT1000S, Leica).

### Immunohistochemistry

Coronal brain sections were permeabilized for 30 min at RT with 0.5% Triton X-100 (#66831, Carl Roth) in PBS and blocked for 30 min at RT with 0.2% BSA (#A7030, Sigma-Aldrich) and 5% (w/v) donkey serum (#017-000-121, Jackson ImmunoResearch) in PBS. Sections were incubated with primary antibodies in 0.2% (w/v) BSA and 0.25% Triton X-100 in PBS at 4°C overnight. The following primary antibodies were used: 1:100 mouse anti-PKCδ (610398, BD Biosciences), 1:500 rabbit anti-cfos (2250S, Cell Signaling Technology, USA), 1:100 rabbit anti-GHSR (PA5-28752, Thermo Fisher Scientific, USA), and 1:100 chicken anti-GFP (green fluorescent protein) (A10262, Thermo Fisher Scientific, USA). Sections were washed three times for 10 min with PBS and incubated for two hours at 4°C with secondary antibodies diluted 1:400 in 0.2% (w/v) BSA and 0.25% Triton X-100 in PBS. The following secondary antibodies were used: donkey anti-rabbit/mouse/chicken Alexa Fluor 488 or Cy3 or Alexa Fluor 647 (anti-rabbit, 711-545-152, 711-165-152, 711-495-152; anti-mouse, 715-545-151, 715-165-151, 715-605-151; anti-chicken, 703-545-155, 703-165-155, 703-605-155, Jackson ImmunoResearch). Sections were washed two times for 10 min with PBS and incubated with DAPI (1:2000) (Sigma-Aldrich) in PBS. After a 15-min wash in PBS, sections were mounted using Fluorescent Mounting Medium (#S3023, Dako).

### Microscopy and image processing

Epifluorescence images were obtained with an upright epifluorescence microscope (Zeiss) with 10×/0.3 objectives (Zeiss). To acquire fluorescence z-stack images, a Leica SP8 confocal microscope equipped with a 20×/0.75 IMM objective (Leica) was used. For full views of the brain slices, a tile scan and automated mosaic merge functions of Leica LAS AF software were used. Images were minimally processed with ImageJ software (NIH) to adjust for brightness and contrast for optimal representation of the data, always keeping the same levels of modifications between control and treated animals.

### Data analysis

Data and statistical analyses were performed using Prism v9.5 (GraphPad, USA) and Excel 2016 (Microsoft, USA). Clampfit software (Molecular Devices, USA) was used to analyze electrophysiological recordings. Softwares used for snRNA-seq were described above. All sample sizes, statistical analyses, and definitions are provided in the figure legends.

## Notes

View/request a protocol for this paper from *Bio-protocol*.
