# Supplementary material for: Transcriptomics reveals amygdala neuron regulation by fasting and ghrelin thereby promoting feeding
Source: Sci Adv. 2023 May 24;9(21):eadf6521. doi: 10.1126/sciadv.adf6521 (PMC10208581; doi:10.1126/sciadv.adf6521)
Supplement: Supplementary file 1 — Figs. S1 to S9 [file sciadv.adf6521_sm.pdf]

Science Advances
AAAS

Supplementary Materials for

Transcriptomics reveals amygdala neuron regulation by fasting and ghrelin thereby promoting feeding

Christian Peters *et al.*

Corresponding author: Rüdiger Klein, ruediger.klein@bi.mpg.de

Sci. Adv. **9**, eadf6521 (2023)
DOI: 10.1126/sciadv.adf6521

This PDF file includes:

Figs. S1 to S9

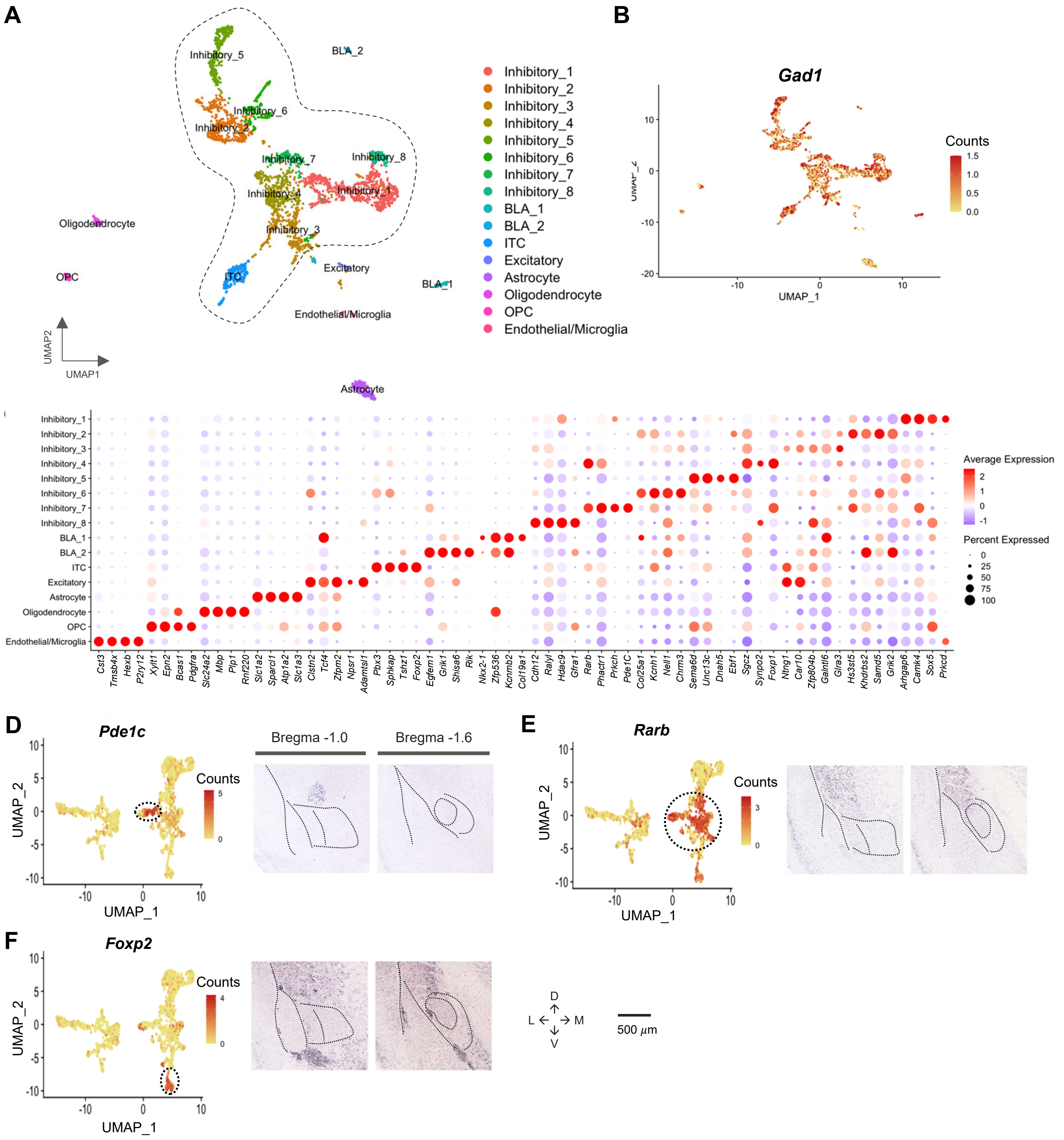

Supplementary Fig. S1 related to Fig. 1: Transcriptomic cell type taxonomy of the mouse central amygdala.
(A) UMAP representation of all sampled cell types including astrocytes, oligodendrocytes, oligodendrocyte progenitor cells (OPCs), vascular endothelial cells/microglia, excitatory neurons, basolateral amygdala (BLA) interneurons, and other inhibitory neurons. The dashed area indicates the cells included in the analysis of Fig. 1B.
(B) Gad1 labels both inhibitory projection and BLA interneurons.
(C) Molecular signatures of clusters by percentage of cells expressing the gene (circle size) and average gene expression (color scale).
(D-F) UMAP plots and RNA ISH images from Allen Developmental Mouse Brain for *Pde1c* (D), *Rarb* (E), and *Foxp2* (F) clusters (indicated by stippled circles). Anterior CeA, bregma -1.0 (left); posterior CeA, bregma -1.6 (right). Scale bar: 500 µm.

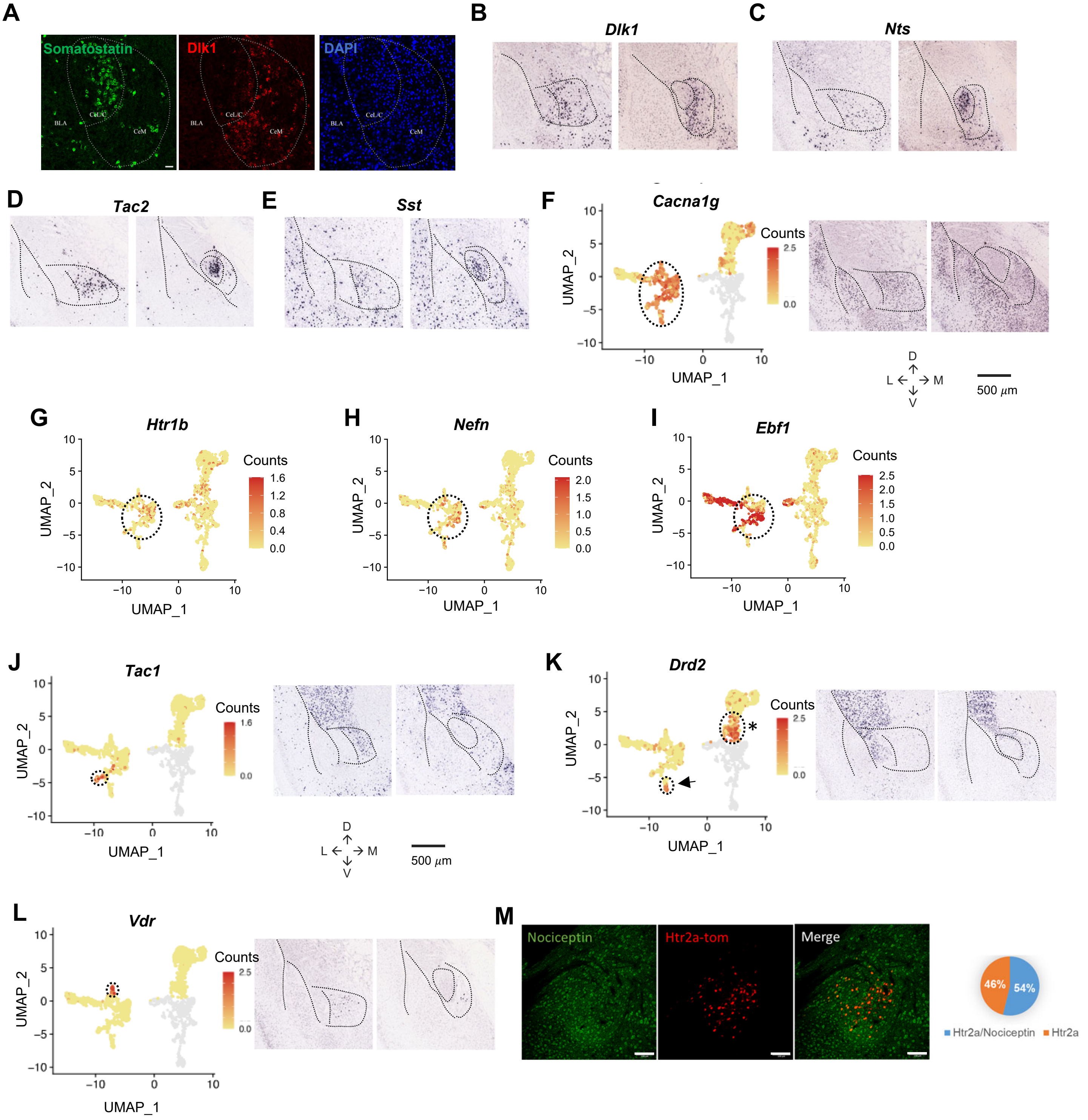

Supplementary Fig. S2 related to Fig. 1: Transcriptomic cell type taxonomy of the mouse central amygdala.
(A) RNA ISH showing Somatostatin (green) and Dlk1 (red) in CeA. CeA subregions are outlined with stippled lines. Scale bar: 30µm.
(B-E) RNA ISH images from Allen Developmental Mouse Brain for Dlk1 (B), Nts (C), Tac2 (D), and Sst (E) cells. Anterior CeA, bregma -1.0 (left); posterior CeA, bregma -1.6 (right). Scale bar : 500 µm.
(F) UMAP plot and RNA ISH images from Allen Developmental Mouse Brain for Cacna1g. CeA, bregma -1.0 (left); posterior CeA, bregma -1.6 (right). Scale bar : 500 µm.
(G-I) UMAP plots for *Htr1b* (G), *Nefm* (H), and *Ebf1* (I) clusters (indicated by stippled circles).
(J-L) UMAP plots and RNA ISH images from Allen Developmental Mouse Brain for *Tac1* (J), *Drd2* (K), and *Vdr* (L) clusters. *Drd2* cluster in CeM is indicated by an arrow. *Drd2* cells in the *Calcrl* cluster are indicated with an asterisk. Anterior CeA, bregma -1.0 (left); posterior CeA, bregma -1.6 (right). Scale bar : 500 µm.
(M) Immunohistochemistry showing the colocalization of CeAHtr2a-tom and Pnoc neurons marked by expression of Nociceptin. The plot on the right show the percentage of colocalization. Scale bar: 250 µm.

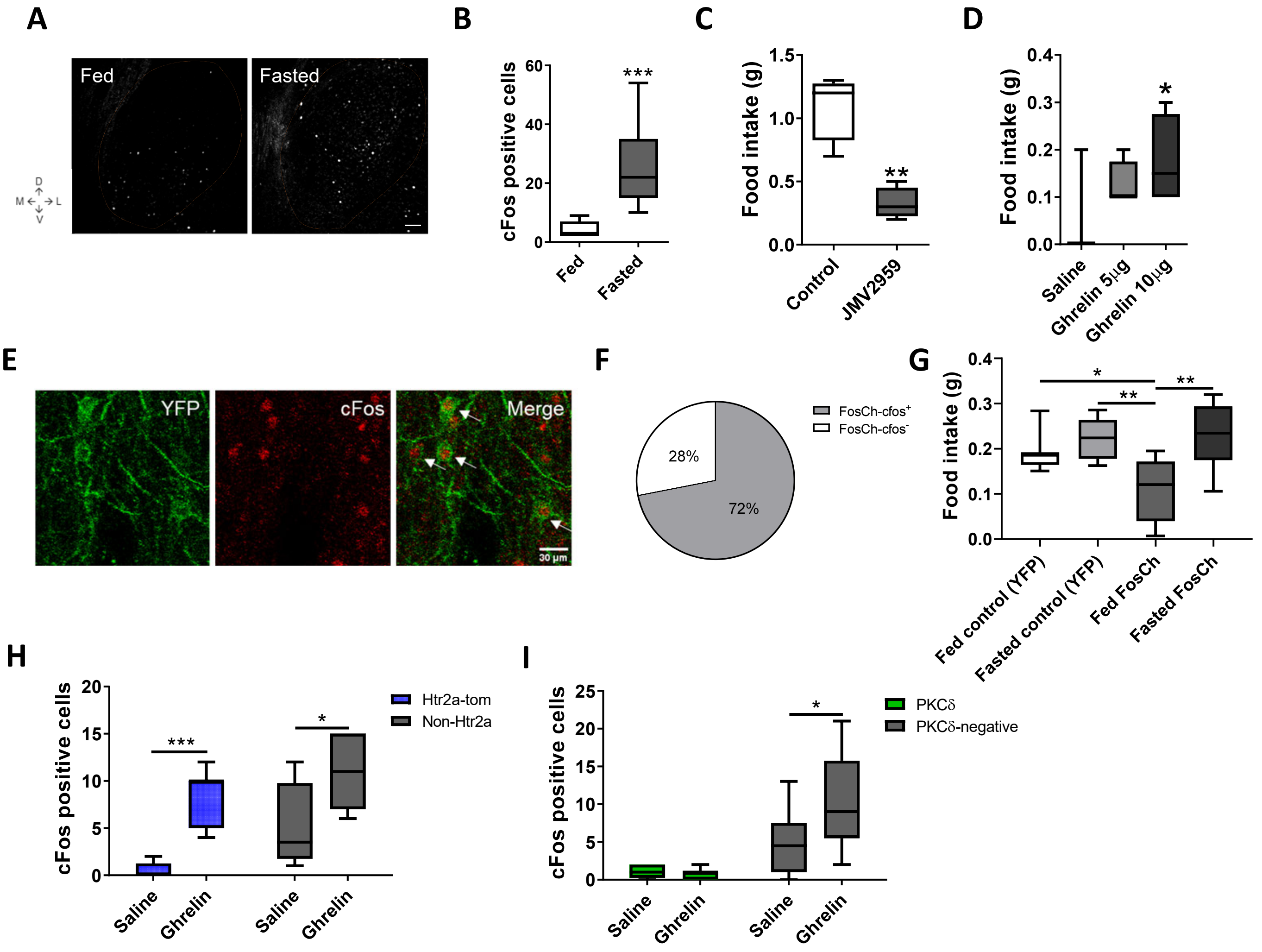

Supplementary Fig. S3 related to Fig. 2: Ghrelin increases food intake and activity of CeA^{Htr2a} neurons.

(A,B) CeA c-Fos staining and quantification in fed and fasted animals (20 h). T-test, ***$p<0.001$, n=8 sections from n=3 animals per group.

(C) Food intake (1 h) of fasted animals (20 h) with or without ghrelin receptor antagonist JMV2959 (250 µg). T-test, *$p<0.05$, **$p<0.01$, n=4 mice per group.

(D) Food intake after intraperitoneal injection of ghrelin (5 or 10 µg). T-test, *$p<0.05$, n=4 mice per group.

(E) Representative immunostaining showing the colocalization (white arrowheads) between YFP (green) and c-Fos (red). Scale bar 30 µm.

(F) Percentage of fosCh positive cells that are also positive for cfos. 19 CeA sections from 4 mice were analysed.

(G) Food intake after 30 min with blue light stimulation (20 Hz) of mice expressing AAV-cFos-hChR2(H134R)-eYFP-Pest virus (FosCh) into CeA. Protocol of feeding assay is describd in Figure 2. T-test, *$p<0.05$, n=6 animals per group. One-way ANOVA, *$p<0.05$, **$p<0.01$, n=6 animals per group.

(H) Numbers of c-Fos positive cells per CeA section among Htr2a and non-Htr2a neurons. Two-way ANOVA, ***$p<0.001$, n=6 sections from n=3 animals per group.

(I) Numbers of c-Fos positive cells among PKCδ and non-PKCδ neurons. Two-way ANOVA, *$p<0.05$, n=8 sections from n=3 animals per group.

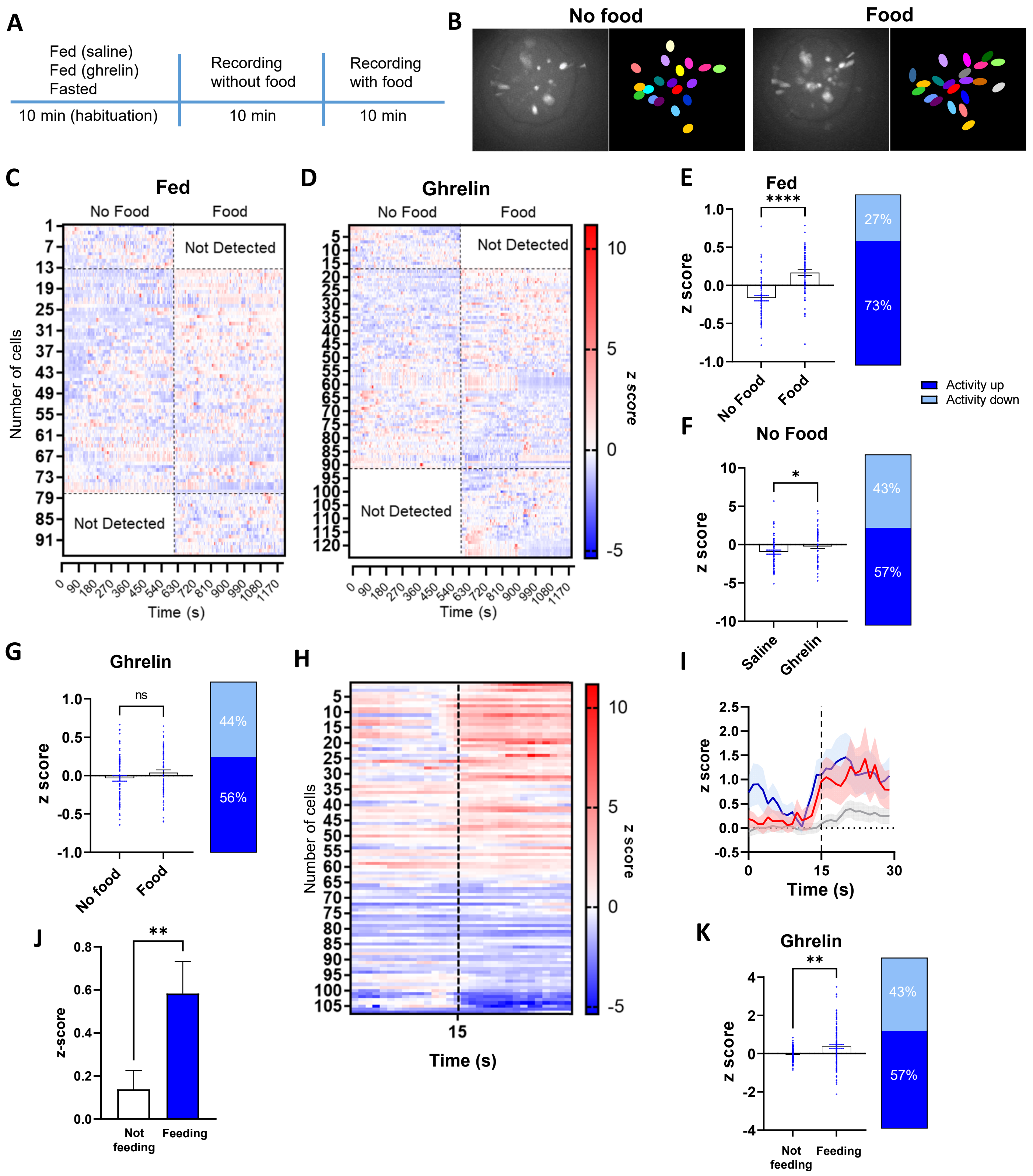

A
Fed (saline)
Fed (ghrelin)
Fasted
Recording without food
Recording with food
10 min (habituation)
10 min
10 min
B
No food
Food
C
Fed
No Food
Food
Not Detected
Not Detected
Number of cells
Time (s)
D
Ghrelin
No Food
Food
Not Detected
Not Detected
z score
Time (s)
E
Fed
****
z score
No Food
Food
27%
73%
Activity up
Activity down
F
No Food
*
z score
Saline
Ghrelin
43%
57%
G
Ghrelin
ns
z score
No food
Food
44%
56%
H
Number of cells
z score
15
Time (s)
I
z score
Time (s)
J
**
z-score
Not feeding
Feeding
K
Ghrelin
**
z score
Not feeding
Feeding
43%
57%

Supplementary Fig. S4: In vivo calcium imaging of CeA^{Htr2a} neurons in response to ghrelin.

(A) Scheme of the behavioural test. I.p. injections were done with 100 µl of saline or ghrelin (10 µg).

(B) Maximum projection images of CeA^{Htr2a}::GCaMP6s-expressing neurons from a representative mouse recorded after i.p. injection of ghrelin with or without food. The images on the right show the corresponding ROIs identifying each cell with a color. Cells identified in both conditions have the same color.

(C,D) Heatmap plots showing the z-scores of CeA^{Htr2a} neuronal activities in fed condition after saline (C) or ghrelin injection (D), without food (first 10 min) or with food (second 10 min).

(E-G) Left: average z-score comparisons between "no food" and "food" conditions, in fed mice (**E**), between saline and ghrelin injections without food (**F**), between "no food" and "food" conditions, in ghrelin injected mice (**G**). Right: percentage of neurons increasing or decreasing the activity in each condition. (E,G) Paired t-test, ****$p<0.0001$, ns, not significant, n=3 mice per group; (F) T-test, *$p<0.05$, n=3 mice per group.

(H) Heat map of showing the z-scores from individual CeA^{Htr2a}::GCaMP6s neurons 15 sec before and during the first eating bout in mice injected with ghrelin. Vertical stippled line indicates eating onset.

(I) Average Ca^{2+} responses of cells from individual animals aligned to the first eating-bout onset after ghrelin injection (n = 3 mice, every line graph corresponds to one mouse, and the shaded areas indicate SEM). Vertical stippled line indicates eating onset.

(J) Average z-score responses from panel I. Wilkoxon test, **$p<0.01$, n=3 mice per group.

(K) Left: average z-score comparison of CeA^{Htr2a} neuronal activities while the animals were feeding or not feeding in mice injected with ghrelin. Right: percentage of neurons more or less active during eating (as in left plots). T-test, **$p<0.01$, n=3 mice per group.

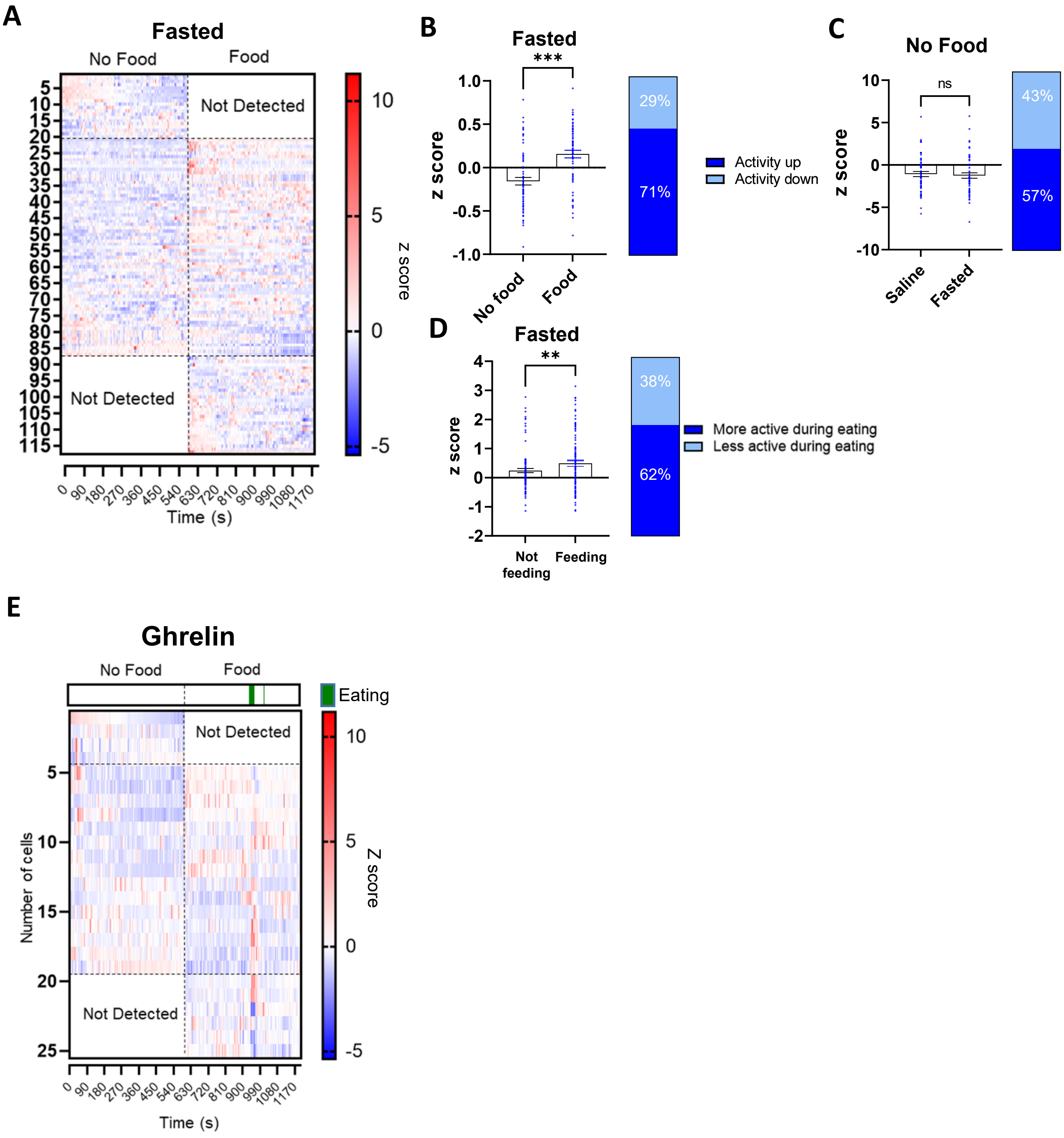

Supplementary figure S5: In vivo calcium imaging showing CeA^{Htr2a} neuronal activity in fasted mice.

(A) Heatmap plots showing the z-score of CeA^{Htr2a} neuronal activity in fasted mice, without food (first 10 min) or with food (second 10 min).

(B) Left: average z-score comparison between "no food" and "food" conditions in fasted mice. Right: percentage of neurons increasing or decreasing the activity in each condition (as in left plots). Wilkoxon test, ****$p<0.0001$, n=3 mice per group.

(C) Left: average z-score comparison between fed ("saline") vs fasted conditions in the absence of food. Right: percentage of neurons increasing or decreasing the activity in each condition (as in left plots). T-test, n=3 mice per group.

(D) Left: average z-score comparison while feeding or not feeding in fasted mice. Right: percentage of neurons more or less active during eating (as in left plots). T-test, **$p<0.01$, n=3 mice per group.

(E) Heatmap plots from a representative animal showing the z-scores of CeA^{Htr2a} neuronal activity injected i.p. with ghrelin (10 µg), without food (first 10 min) or with food (second 10 min). Green bars indicate the times in which the mice were feeding.

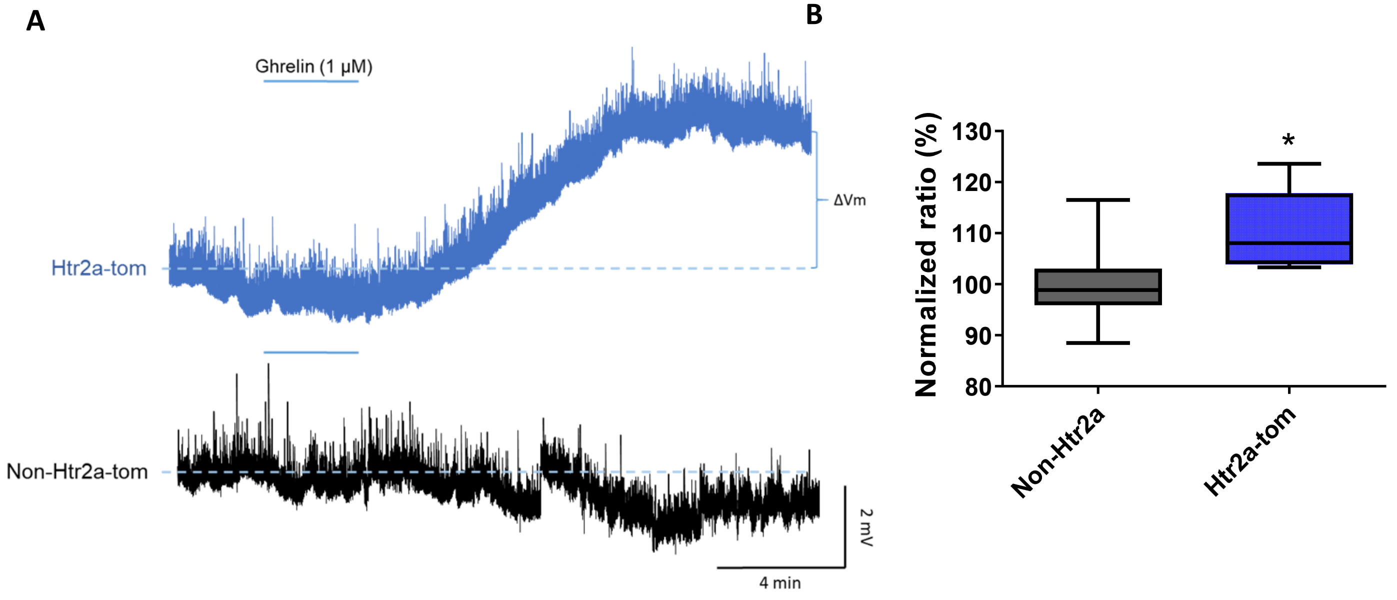

Supplementary Fig. S6 related to Fig. 3: Ghrelin excites CeA^{Htr2a} neurons.

(A) Whole-cell current-clamp recordings of CeA^{Htr2a} and CeA$^{Non\text{-}Htr2a}$ neurons, showing that ghrelin depolarized only CeA^{Htr2a} neurons after 3 min of ghrelin perfusion (1 µM).

(B) Normalized ratios of the voltage difference comparing CeA^{Htr2a} and CeA$^{Non\text{-}Htr2a}$ neurons in response to 1 µM ghrelin.

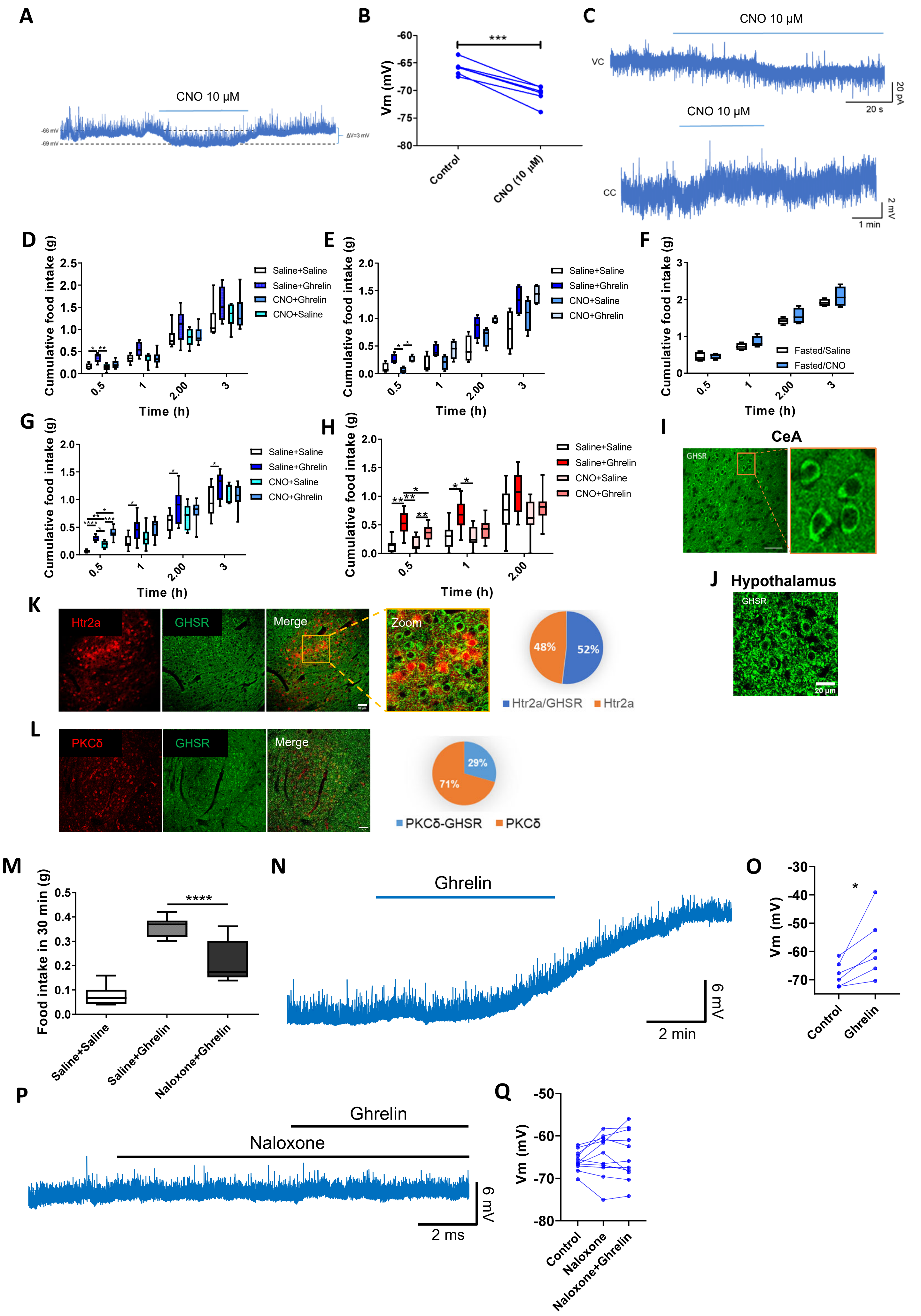

A
CNO 10 μM
-66 mV
-69 mV
ΔV=3 mV
B
***
Vm (mV)
Control
CNO (10 μM)
C
CNO 10 μM
VC
20 pA
20 s
CC
2 mV
1 min
D
Cumulative food intake (g)
Time (h)
Saline+Saline
Saline+Ghrelin
CNO+Ghrelin
CNO+Saline
E
Saline+Saline
Saline+Ghrelin
CNO+Saline
CNO+Ghrelin
F
Fasted/Saline
Fasted/CNO
G
H
I
CeA
GHSR
J
Hypothalamus
GHSR
20 μm
K
Htr2a
GHSR
Merge
Zoom
48%
52%
Htr2a/GHSR
Htr2a
L
PKCδ
GHSR
Merge
29%
71%
PKCδ-GHSR
PKCδ
M
Food intake in 30 min (g)
****
Saline+Saline
Saline+Ghrelin
Naloxone+Ghrelin
N
Ghrelin
6 mV
2 min
O
Vm (mV)
*
Control
Ghrelin
P
Ghrelin
Naloxone
6 mV
2 ms
Q
Vm (mV)
Control
Naloxone
Naloxone+Ghrelin

Supplementary Fig. S7 related to Fig. 4: Ghrelin increases feeding through CeA^{Htr2a} neurons.

(A) Representative current-clamp slice recording perfusing CNO (10 µM) in CeA^{Htr2a} neurons expressing pAAV-hSyn-DIO-hM4D(Gi)-mCherry virus.

(B) Plot showing the hyperpolarization of the membrane potential produced by CNO (10 µM) in mice from panel A.

(C) Voltage (top) and current (bottom) clamp recordings showing the excitation of CeA^{Htr2a} neurons after CNO (10 µM) application in mice expressing pAAV-hSyn-DIO-hM3Dq-mCherry virus.

(D) Cumulative food intake of satiated Htr2-cre animals expressing pAAV-hSyn-DIO-hM4D(Gi)-mCherry virus in CeA, after i.p. injections of saline, CNO (0.4 mg/Kg) and ghrelin (10 µg). Two-way ANOVA, *$p<0.05$, **$p<0.01$, n=6 mice per group

(E) Cumulative food intake of satiated Htr2a-cre animals expressing pAAV-hSyn-DIO-mCherry virus in CeA, after i.p. injections of saline, CNO (0.4 mg/Kg) and ghrelin (10 µg). Two-way ANOVA, *$p<0.05$, **$p<0.01$, n=4 mice per group

(F) Cumulative food intake of fasted Htr2a-cre animals expressing pAAV-hSyn-DIO-hM4D(Gi)-mCherry virus in CeA, after i.p. injections of saline or CNO (0.4 mg/Kg).

(G) Cumulative food intake of satiated Htr2a-cre animals expressing the excitatory DREADD hM3Dq (pAAV-hSyn-DIO-hM3Dq-mCherry) virus in CeA, after i.p. injections of saline, CNO (1 mg/Kg) and ghrelin (10 µg). Two-way ANOVA, *$p<0.05$, **$p<0.01$, n=8 mice per group

(H) Cumulative food intake in satiated CeA→PBN projectors mice expressing hM4D(Gi) using a combination of i.p. injections with saline, CNO, and ghrelin. Two-way ANOVA, *$p<0.05$, **$p<0.01$, n=8 mice per group.

(I-L) Immunostainings for GHSR in wild-type mice CeA (I),in hypothalamus (J), in Htr2a-Cre;tdTomato mice (CeA) (K), and in combination with PKCδ stainings (CeA) (L). The circular plots show the percentage of colocalization between GHSR and Htr2a or PKCδ. Scale bars represent 50 µm.

(M) Food intake (30 min) in satiated mice after i.p. injections of saline, the opioid receptor antagonist naloxone (100 µg) and ghrelin (10 µg). One-way ANOVA, ****$p<0.0001$, n=8 animals per group.

(N) Whole-cell current-clamp recordings of CeA$^{Htr2a\text{-}tom}$ showing that ghrelin depolarized Htr2a-tom neurons after 6 min of ghrelin perfusion (100 nM).

(O) Quantification of the membrane potentials before and after ghrelin perfusions from panel M. Paired t-test, *$p<0.05$, n=6.

(P) Whole-cell current-clamp recordings of CeA$^{Htr2a\text{-}tom}$ after 6 min Naloxone perfusion (1 µM) and 6 min with Naloxone (1 µM) + Ghrelin (100 nM).

(Q) Quantification of the membrane potentials from panel D. One-way ANOVA, n=11.

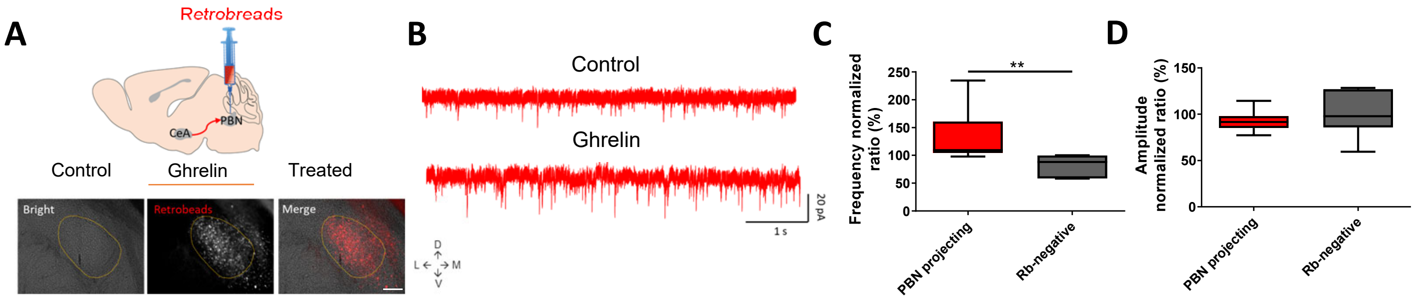

Supplementary Fig. S8 related to Fig. 5: CeA→PBN projectors increase activity after ghrelin perfusion.

(A) Retrobeads (red) were stereotactically injected into PBN and retrogradely transported into the CeA by CeA neurons projecting to PBN (bottom histology). Scale bar represents 250 µm.

(B) Representative sEPSC before (control) and after 3 min of ghrelin perfusion (ghrelin).

(C, D) Frequency and amplitude normalized ratio comparison between PBN-projecting and retrobeads-negative (non-PBN-projecting) neurons. Paired t-test, **p<0.01, n=3 mice per group.

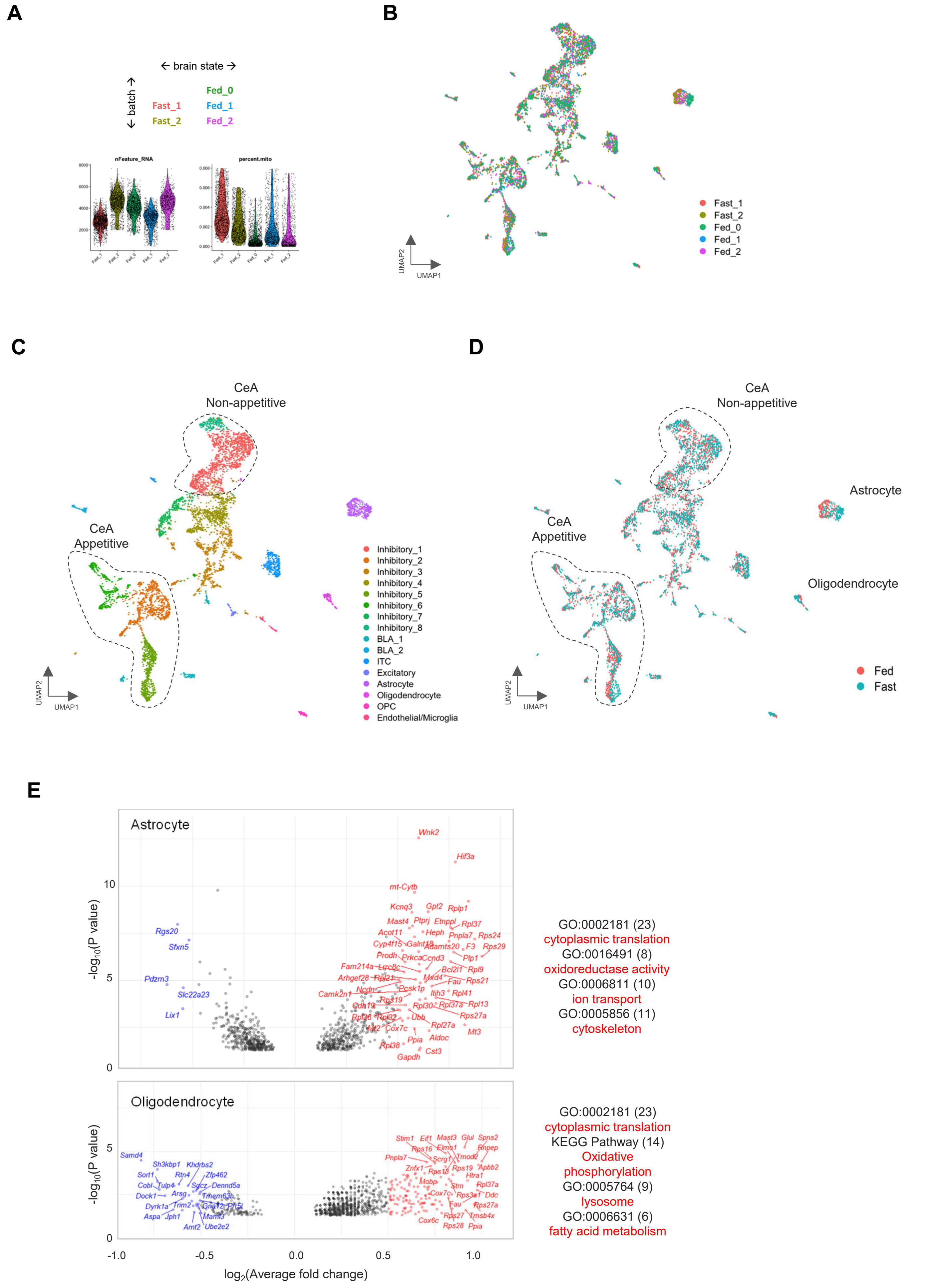
A
← brain state →
← batch →
Fed_0
Fast_1
Fed_1
Fast_2
Fed_2
nFeature_RNA
percent.mito
B
Fast_1
Fast_2
Fed_0
Fed_1
Fed_2
UMAP2
UMAP1
C
CeA
Non-appetitive
CeA
Appetitive
Inhibitory_1
Inhibitory_2
Inhibitory_3
Inhibitory_4
Inhibitory_5
Inhibitory_6
Inhibitory_7
Inhibitory_8
BLA_1
BLA_2
ITC
Excitatory
Astrocyte
Oligodendrocyte
OPC
Endothelial/Microglia
UMAP2
UMAP1
D
CeA
Non-appetitive
Astrocyte
CeA
Appetitive
Oligodendrocyte
Fed
Fast
UMAP2
UMAP1
E
Astrocyte
Oligodendrocyte
-log10(P value)
log2(Average fold change)
GO:0002181 (23)
cytoplasmic translation
GO:0016491 (8)
oxidoreductase activity
GO:0006811 (10)
ion transport
GO:0005856 (11)
cytoskeleton
GO:0002181 (23)
cytoplasmic translation
KEGG Pathway (14)
Oxidative
phosphorylation
GO:0005764 (9)
lysosome
GO:0006631 (6)
fatty acid metabolism

Supplementary Fig. S9 related to Fig. 7: Transcriptomic changes in sampled CeA cell populations.

(A) Experimental design for acquiring brain state-related snRNAseq datasets (top) and the quality metric for each dataset (bottom). Fed_1 and Fast_1 were prepared in the same batch of single nuclei preparation and library construction, as were Fed_2 and Fast_2. Fed_0 was another batch of biological replicates that only had the sample of satiated mice. (See also Fig. 2B and methods). The median value of genes per nuclei (nFeature_RNA) detected was 3636 (range 3000-5000), while the ratio of mitochondrial RNA (percent.mito) content per nuclei was below 0.008, suggesting low debris content.

(B-D) UMAP representations of all single nuclei from 5 datasets, colored by cell clusters (B) or dataset origin (C).

(E) Volcano plots showing the identified differential expressed (DE) genes that related to brain states in astrocyte (D) and oligodendrocyte (E). Criteria for DE gene: Wilcoxon test $p<0.001$, log2(Average fold change)>0.25. Genes with log2(Average fold change)>0.5 higher in the Fasted group are labeled in red, while lower than that in blue. Official gene symbols are shown with the highest fold changes.
